# Incident cardiovascular events and imaging phenotypes in UK Biobank participants with past cancer

**DOI:** 10.1136/heartjnl-2022-321888

**Published:** 2023-04-18

**Authors:** Zahra Raisi-Estabragh, Jackie Cooper, Celeste McCracken, Emma J Crosbie, Fiona M Walter, Charlotte H Manisty, John Robson, Mamas A Mamas, Nicholas C Harvey, Stefan Neubauer, Steffen E Petersen

**Affiliations:** 1 William Harvey Research Institute, NIHR Barts Biomedical Research Centre, Queen Mary University of London, London, UK; 2 Barts Heart Centre, St Bartholomew’s Hospital, Barts Health NHS Trust, London, UK; 3 Division of Cardiovascular Medicine, Radcliffe Department of Medicine, University of Oxford, NIHR Oxford Biomedical Research Centre, Oxford University Hospitals NHS Foundation Trust, Oxford, UK; 4 Division of Cancer Sciences, Faculty of Biology, Medicine and Health, University of Manchester, Manchester, UK; 5 Department of Obstetrics and Gynaecology, St Mary’s Hospital, Manchester University NHS Foundation Trust, Manchester Academic Health Science Centre, Manchester, UK; 6 Wolfson Institute of Population Health, Queen Mary University of London, London, UK; 7 Department of Public Health and Primary Care, University of Cambridge, Cambridge, UK; 8 Institute of Cardiovascular Science, University College London, London, UK; 9 Institute of Population Health, Manchester University, manchester, UK; 10 Keele Cardiovascular Research Group, Keele University, Keele, UK; 11 MRC Lifecourse Epidemiology Centre, University of Southampton, Southampton, UK; 12 NIHR Southampton Biomedical Research Centre, University of Southampton and University Hospital Southampton NHS Foundation Trust, Southampton, UK; 13 Health Data Research UK, London, UK; 14 Alan Turing Institute, London, UK

**Keywords:** epidemiology, magnetic resonance imaging, epidemiology

## Abstract

**Objectives:**

To evaluate incident cardiovascular outcomes and imaging phenotypes in UK Biobank participants with previous cancer.

**Methods:**

Cancer and cardiovascular disease (CVD) diagnoses were ascertained using health record linkage. Participants with cancer history (breast, lung, prostate, colorectal, uterus, haematological) were propensity matched on vascular risk factors to non-cancer controls. Competing risk regression was used to calculate subdistribution HRs (SHRs) for associations of cancer history with incident CVD (ischaemic heart disease (IHD), non-ischaemic cardiomyopathy (NICM), heart failure (HF), atrial fibrillation/flutter, stroke, pericarditis, venous thromboembolism (VTE)) and mortality outcomes (any CVD, IHD, HF/NICM, stroke, hypertensive disease) over 11.8±1.7 years of prospective follow-up. Linear regression was used to assess associations of cancer history with left ventricular (LV) and left atrial metrics.

**Results:**

We studied 18 714 participants (67% women, age: 62 (IQR: 57–66) years, 97% white ethnicities) with cancer history, including 1354 individuals with cardiovascular magnetic resonance. Participants with cancer had high burden of vascular risk factors and prevalent CVDs. Haematological cancer was associated with increased risk of all incident CVDs considered (SHRs: 1.92–3.56), larger chamber volumes, lower ejection fractions, and poorer LV strain. Breast cancer was associated with increased risk of selected CVDs (NICM, HF, pericarditis and VTE; SHRs: 1.34–2.03), HF/NICM death, hypertensive disease death, lower LV ejection fraction, and lower LV global function index. Lung cancer was associated with increased risk of pericarditis, HF, and CVD death. Prostate cancer was linked to increased VTE risk.

**Conclusions:**

Cancer history is linked to increased risk of incident CVDs and adverse cardiac remodelling independent of shared vascular risk factors.

**WHAT IS ALREADY KNOWN ON THIS TOPIC**

- Few studies have reported associations of past cancer with incident cardiovascular outcomes in large population-based cohorts, and none have included cardiovascular imaging.

**WHAT THIS STUDY ADDS**

- We studied 18 714 UK Biobank participants with history of six common cancers and an equal number of non-cancer comparators propensity matched on vascular risk factors. Our results demonstrate association of cancer history with increased risk of a wide range of incident cardiovascular disease and mortality outcomes over 12 years of prospective follow-up. In participants with cardiovascular magnetic resonance (n=1354), cancer history was linked to adverse cardiac remodelling. The greatest range and magnitude of risk was observed in those with past breast and haematological cancers.

**HOW THIS STUDY MIGHT AFFECT RESEARCH, PRACTICE OR POLICY**

- People with past cancer have heightened cardiovascular risk, which appears independent of vascular risk factors and persists several years after initial cancer diagnosis. This study highlights the specific cardiovascular care needs of patients with cancer and supports consideration of cancer-specific exposures in cardiovascular risk stratification.

## Introduction

Patients with cancer history represent a growing cohort at heightened cardiovascular risk, attributed to shared vascular risk factors, cardiotoxicities of cancer therapies, and biological processes related to the cancer itself.^1 2^ There is differential propensity to cardiovascular disease (CVD) across cancer sites, reflecting variation in these risk exposures.^3 4^

Existing work indicates highest risk of cardiovascular complications to be in the first year after cancer diagnosis.^5^ Few researchers have examined longer term cancer-specific cardiovascular risk in population samples. Such analyses are important for informing cardiovascular risk stratification, surveillance, and treatment of patients with past cancer.

Cardiovascular imaging has a key role in detecting subclinical cardiotoxicity. However, associations of cancer with cardiovascular remodelling in population cohorts have not been previously reported.

We evaluated cardiovascular health in 18 714 UK Biobank participants with previous cancer, characterising disease and risk factor burden, incident disease and mortality outcomes, and cardiovascular remodelling patterns.

## Methods

### Setting and study population

The UK Biobank includes over 500 000 participants aged 40–69 years, characterised in detail at baseline recruitment (2006–2010).^6^ Incident health events are prospectively tracked through extensive health record linkages (Hospital Episode Statistics (HES), cancer register, death register). The UK Biobank Imaging Study, which includes cardiovascular magnetic resonance (CMR), is underway and aims to scan 100 000 of the original participants.

### Ascertainment of cancer history

Cancer history was ascertained from cancer registry and HES records (online supplemental table 1). We created six categories (lung, breast, prostate, haematological, uterus, colorectal) to capture the most common cancer sites.^7^ The primary cancer site was defined from the first code for cancer in any of the linked databases.

10.1136/heartjnl-2022-321888.supp1 Supplementary data

### Ascertainment of incident cardiovascular outcomes

We defined incident CVD (ischaemic heart disease (IHD), stroke, atrial fibrillation (AF)/flutter, heart failure (HF), non-ischaemic cardiomyopathies (NICM), venous thromboembolism (VTE; deep vein thrombosis (DVT), pulmonary embolus (PE)), pericarditis) and mortality outcomes (IHD, stroke, hypertensive diseases, HF or NICMs) using HES and death registration records (online supplemental table 2).

### CMR acquisition and analysis

CMR scans were performed according to predefined protocols and analysed using automated pipelines.^8–10^ These are research scans without any clinical indication. The following metrics were included: left ventricular (LV) end-diastolic volume (LVEDV), LV ejection fraction (LVEF), LV global function index (LVGFI), LV global longitudinal strain (GLS), left atrial (LA) maximum volume (LAV) and LA ejection fraction (LAEF).

### Statistical analysis

Statistical analysis was performed using R studio V.4.1.0 (https://www.R-project.org/) and Stata V.17.^11^ Baseline characteristics are presented as number (percentage) for categorical variables, mean (SD) for normally distributed continuous variables and median (IQR) for non-normally distributed continuous variables. A propensity matched non-cancer comparator cohort was created with a priori selection of covariates (online supplemental figure 1, tables 3 and 4). Comparators were participants without record of cancer at baseline. Each cancer exposed participant was matched to one non-exposed participant using nearest neighbour propensity score matching on 20 predefined baseline covariates. Pairs were discarded if no matching participant had logit propensity score within 0.2 SDs of the case.^12^ Balance of covariates was assessed in the unmatched and matched samples using the standardised mean difference between exposed and non-exposed groups (online supplemental figure 2). Missing data values were imputed using single centre imputation from the multiple chained equation algorithm.

Competing risks regression was used to calculate subdistribution HRs (SHR) and 95% CIs for the association of cancer history at baseline with incident disease and mortality outcomes. Participants with the outcome of interest at baseline were excluded from analyses for that outcome (but included in analyses of other outcomes). Incident events were first occurrence of the outcome after baseline. Prevalent events were conditions present at baseline. The censor date was 26 March 2021, providing mean prospective follow-up of 11.8±1.7 years. We performed sensitivity analyses using cause-specific Cox regression, limiting to cases with complete data (no imputation), and to cancers diagnosed within 5 years prior to baseline. Given possible heterogeneities within the haematological cancer category, we examined associations with incident outcomes within its subcategories (lymphoma, leukaemia, myeloma). We tested for interaction of cancer exposure with time by defining time from cancer diagnosis to baseline for cases and assigning the same time to their matched controls.

Linear regression was used to estimate association of cancer exposure with each CMR metric, reporting standardised beta coefficients, 95% CIs, and p values. For this analysis, cancer status was ascertained at imaging (any cancer diagnosis had been established prior to imaging). The samples all matched well on overall propensity score; individual covariates that were less well matched were included as covariates in final models, as per Nguyen *et al* (online supplemental figure 3).^13^ We repeated the analysis excluding individuals with CVD at time of imaging. A two-sided significance level of 0.05 was used for all comparisons.

## Results

### Baseline characteristics

We analysed 18 714 participants with past cancer (online supplemental figure 4). Smoking was most common in those with lung (82.9%), colorectal (54.4%) and prostate (53.0%) cancer (table 1). Diabetes was most common in lung (9.9%), uterine (9.5%), and colorectal (8.8%) cancer. The highest rates of hypertension were in prostate (45.6%), colorectal (39.5%), and uterine (38.4%) cancer. Individuals with uterine cancer had the highest average body mass index. Among those with cancer, 17.6% had pre-existing CVD (table 2).

**Table 1:** Baseline participant characteristics

	Cases	Controls	Breast	Lung	Prostate	Colorectal	Uterus	Haem
N	18 714	18 714	9531*	313	3291	2412	937	2230
Age	62 (57–66)	62 (57–66)	61 (56–65)	62 (58–66)	65 (62–67)	63 (59–66)	63 (59–66)	60 (53–65)
Men	6095 (32.6)	6095 (32.6)	0 (0)	170 (54.3)	3291 (100)	1383 (57.3)	0 (0)	1251 (56.1)
Women	12 619 (67.4)	12 619 (67.4)	9531 (100)	143 (45.7)	0 (0)	1029 (42.7)	937 (100)	979 (43.9)
White ethnicity	18 002 (96.7)	18 025 (96.7)	9201 (96.9)	301 (96.2)	3143 (96.1)	2324 (96.6)	910 (97.5)	2146 (96.7)
BAME	617 (3.3)	611 (3.3)	299 (3.2)	12 (3.8)	129 (3.9)	81 (3.4)	23 (2.5)	73 (3.3)
Townsend score	−2.3 (−3.7 to 0.3)	−2.3 (−3.7 to 0.3)	−2.3 (−3.7 to 0.2)	−0.7 (−3.3 to 2.5)	−2.4 (−3.8 to −0.1)	−2.2 (−3.7 to 0.4)	−2.2 (−3.6 to 0.0)	−2.2 (−3.6 to 0.5)
Degree or professional qualification	8329 (45.5)	8300 (45.4)	4259 (45.5)	96 (32.1)	1513 (47.1)	1022 (43.3)	382 (42.0)	1057 (48.5)
SBP (mm Hg)	140.2±19.2	140.1±19.1	138.5±19.4	137.7±19.3	145.0±17.8	142.6±19.2	141.2±18.7	137.5±18.9
DBP (mm Hg)	82.0±10.1	82.0±10.0	81.4±9.9	81.5±11.2	84.0±9.9	82.6±10.1	82.1±9.6	81.1±10.6
HR (bpm)	70.5 (63.5–78.5)	70(63-78)	71.5(65-79)	75 (67–83.5)	67.5 (60.5–75.5)	69.5 (62.5–77.5)	71(64-78)	70.5(63-80)
BMI (kg/m^2^)	26.8 (24.2–30.0)	26.7 (24.1–29.9)	26.4 (23.7–29.7)	26.7 (24.3–30.1)	27.4 (25.1–30.0)	27.2 (24.7–30.2)	28.4 (24.7–33.7)	26.8 (24.2–30.0)
Physical activity (METS/week)	1695 (754–3426)	1742 (782–3471)	1695 (777–3336)	1175 (375–2799)	1874 (817–3848)	1626 (704–3412)	1624 (710–3506)	1578 (693–3279)
Ever smoked	8909 (48.0)	9141 (49.2)	4225 (44.6)	257 (82.9)	1725 (53.0)	1304 (54.4)	342 (36.8)	1056 (47.6)
HbA1c (mmol/mol)	36 (33.5–38.7)	35.9 (33.4–38.5)	36 (33.7–38.5)	37 (34.1–39.7)	36 (33.4–38.6)	36 (33.4–39.1)	36.4 (34.1–39.2)	35.5 (32.8–38.4)
Random glucose (mmol/L)	5.0 (4.7–5.4)	5.0 (4.6–5.4)	5.0 (4.7–5.4)	4.9 (4.6–5.4)	5.0 (4.7–5.5)	5.1 (4.7–5.5)	5.0 (4.7–5.5)	5.0 (4.6–5.4)
Total cholesterol (mmol/L)	5.8±1.2	5.8±1.2	6.0±1.2	5.6±1.3	5.4±1.1	5.6±1.2	5.9±1.2	5.6±1.2
HDL (mmol/L)	1.4 (1.2–1.7)	1.4 (1.2–1.7)	1.6 (1.3–1.8)	1.3 (1.1–1.6)	1.3 (1.1–1.5)	1.4 (1.1–1.7)	1.5 (1.3–1.7)	1.3 (1.1–1.6)
LDL (mmol/L)	3.5 (2.9–4.2)	3.6 (2.9–4.2)	3.6 (3.0–4.3)	3.4 (2.8–4.1)	3.4 (2.8–4.0)	3.4 (2.8–4.1)	3.6 (3.0–4.3)	3.5 (2.9–4.1)
Triglyceride level (mmol/L)	1.6 (1.1–2.2)	1.5 (1.1–2.2)	1.5 (1.1–2.1)	1.7 (1.2–2.3)	1.7 (1.2–2.4	1.7 (1.2–2.4)	1.6 (1.2–2.2)	1.6 (1.1–2.4)
Diabetes	1222 (6.5)	1238 (6.6)	463 (4.9)	31 (9.9)	264 (8.0)	211 (8.8)	89 (9.5)	164 (7.4)
Hypertension	6421 (34.3)	6443 (34.4)	2761 (29.0)	108 (34.5)	1499 (45.6)	953 (39.5)	360 (38.4)	740 (33.2)
High cholesterol	5659 (30.2)	5627 (30.1)	2272 (23.8)	115 (36.7)	1431 (43.5)	882 (36.6)	304 (32.4)	655 (29.4)

Count variables are shown as N (%). Continuous variables are shown as mean±SD or median (IQR) if skewed.

*39 males excluded

BAME, black, Asian and minority ethnic; BMI, body mass index; DBP, diastolic blood pressure; Haem, haematological; HbA1c, glycated haemoglobin; HDL, high density lipoprotein; LDL, low density lipoprotein; MET, metabolic equivalent; SBP, systolic blood pressure.

**Table 2:** Prevalent and incident cardiovascular diseases and mortality

	Cases	Controls	Breast	Lung	Prostate	Colorectal	Uterus	Haem
N (total)	18 714	18 714	9531	313	3291	2412	937	2230
Prevalent CVDs (N, %)	3289 (17.6)	2856 (15.3)	1119 (11.7)	116 (37.1)	805 (24.5)	554 (23.0)	121 (12.9)	574 (25.7)
IHD	1238 (6.6)	1286 (6.9)	348 (3.7)	45 (14.4)	375 (11.4)	222 (9.2)	45 (4.8)	203 (9.1)
NICM	52 (0.3)	33 (0.2)	21 (0.2)	1 (0.3)	11 (0.3)	7 (0.3)	2 (0.2)	10 (0.4)
HF	152 (0.8)	97 (0.5)	44 (0.5)	7 (2.2)	38 (1.2)	21 (0.9)	4 (0.4)	38 (1.7)
AF/flutter	431 (2.3)	394 (2.1)	111 (1.2)	23 (7.3)	138 (4.2)	71 (2.9)	14 (1.5)	74 (3.3)
Stroke	426 (2.3)	448 (2.4)	160 (1.7)	18 (5.8)	100 (3.0)	63 (2.6)	15 (1.6)	70 (3.1)
Pericarditis	35 (0.2)	22 (0.1)	17 (0.2)	1 (0.3)	7 (0.2)	4 (0.2)	0	6 (0.3)
VTE (DVT/PE)	955 (5.1)	576 (3.1)	418 (4.4)	21 (6.7)	136 (4.1)	166 (6.9)	41 (4.4)	173 (7.8)
Incident CVDs (N, %) (rate per 1000 person-years)	5753 (30.7) (21.5)	4594 (24.5) (16.3)	2130 (22.3) (14.7)	155 (49.5) (32.3)	1335 (40.6) (27.6)	803 (33.3) (22.8)	250 (26.7) (15.9)	1080 (48.4) (30.7)
IHD	1584 (8.5) (7.8)	1425 (7.6) (7.0)	560 (5.9) (5.5)	40 (12.8) (19.4)	385 (11.7) (12.3)	245 (10.2) (20.8)	68 (7.3) (6.9)	286 (12.8) (14.1)
NICM	225 (1.2) (1.0)	134 (0.7) (0.6)	90 (0.9) (0.8)	2 (0.6) (0.7)	38 (1.2) (1.1)	31 (1.3) (1.2)	7 (0.7) (0.6)	57 (2.6) (2.5)
HF	950 (5.1) (4.3)	705 (3.8) (3.2)	337 (3.5) (3.2)	32 (10.2) (12.5)	205 (6.2) (5.8)	107 (4.4) (4.2)	42 (4.5) (3.9)	227 (10.2) (10.0)
AF/flutter	1539 (8.2) (7.2)	1317 (7.0) (6.1)	555 (5.8) (5.4)	38 (12.1) (15.4)	382 (11.6) (11.6)	236 (9.8) (9.7)	69 (7.4) (6.3)	259 (11.6) (11.8)
Stroke	590 (3.2) (2.7)	477 (2.5) (2.2)	211 (2.2) (2.0)	16 (5.1) (6.6)	148 (4.5) (4.4)	83 (3.4) (3.3)	30 (3.2) (2.8)	102 (4.6) (4.6)
Pericarditis	188 (1.0) (0.8)	94 (0.5) (0.4)	75 (0.8) (0.7)	12 (3.8) (4.8)	28 (0.9) (0.8)	19 (0.8) (0.7)	7 (0.7) (0.6)	47 (2.1) (2.0)
VTE (DVT/PE)	677 (3.6) (3.4)	442 (2.4) (2.1)	302 (3.2) (2.9)	15 (4.8) (5.8)	149 (4.5) (4.3)	82 (3.4) (3.4)	27 (2.9) (2.7)	102 (4.6) (4.7)
Mortality outcomes (N, %) (rate per 1000 person-years)	3514 (18.8) (17.0)	1582 (8.5) (7.2)	1454 (15.3) (13.5)	160 (51.1) (59.0)	683 (20.8) (18.9)	499 (20.7) (19.1)	113 (12.1) (10.4)	605 (27.1) (25.7)
Any CVD	287 (1.5) (1.4)	265 (1.4) (1.2)	74 (0.8) (0.7)	17 (5.4) (6.3)	83 (2.5) (2.3)	54 (2.2) (2.1)	12 (1.3) (1.1)	47 (2.1) (2.0)
IHD	154 (0.8) (0.7)	160 (0.9) (0.7)	24 (0.3) (0.2)	14 (4.5) (5.2)	53 (1.6) (1.5)	34 (1.4) (1.3)	3 (0.3) (0.3)	26 (1.2) (1.1)
HF/NICM	37 (0.2) (0.2)	17 (0.1) (0.1)	17 (0.2) (0.2)	0	7 (0.2) (0.2)	5 (0.2) (0.2)	3 (0.3) (0.3)	5 (0.2) (0.2)
Stroke	65 (0.3) (0.3)	60 (0.3) (0.3)	21 (0.2) (0.2)	2 (0.6) (0.7)	16 (0.5) (0.4)	11 (0.5) (0.4)	5 (0.5) (0.5)	10 (0.4) (0.4)
Hypertensive diseases	21 (0.1) (0.1)	9 (0.1) (0.04)	8 (0.1) (0.1)	0	5 (0.2) (0.1)	3 (0.1) (0.1)	2 (0.2) (0.2)	3 (0.1) (0.1)

Figures are numbers of participant with each condition/outcome. Percentages are shown in brackets with denominator taken as the total number of participants in each category (‘total’ row). Prevalent CVDs were present at baseline recruitment. Incident CVDs represent first occurrence of the condition after baseline.

AF, atrial fibrillation; CVD, cardiovascular disease; DVT, deep vein thrombosis; Haem, haematological; HF, heart failure; IHD, ischaemic heart disease; NICM, non-ischaemic cardiomyopathies; PE, pulmonary embolism; VTE, venous thromboembolism.

### Incident events

Almost one-third of participants with cancer developed one of the incident CVDs (table 2). The highest rates of incident CVD were in participants with lung (49.5%), haematological (48.4%), and prostate (40.6%) cancer. Incident IHD, AF/flutter and HF were the top three incident CVDs across all cancers. Over the study period, 18.8% of participants with cancer died compared with 8.5% of controls. In those with cancer, 8.2% (287/3514) of deaths were primary cardiovascular deaths.

### Breast cancer

Among participants with breast cancer, 22.3% (2130/9531) developed one of the incident CVDs considered and 15.3% (1454/9531) died. The most common incident CVDs were IHD (5.9%), AF/flutter (5.8%), HF (3.5%), VTE (3.2%) and stroke (2.2%). NICMs occurred in 0.9% and pericarditis in 0.8% of participants with breast cancer. A total of 5.1% (74/1454) of all deaths were primary cardiovascular deaths. The most common causes of CVD death were stroke and IHD.

**Figure 1:**
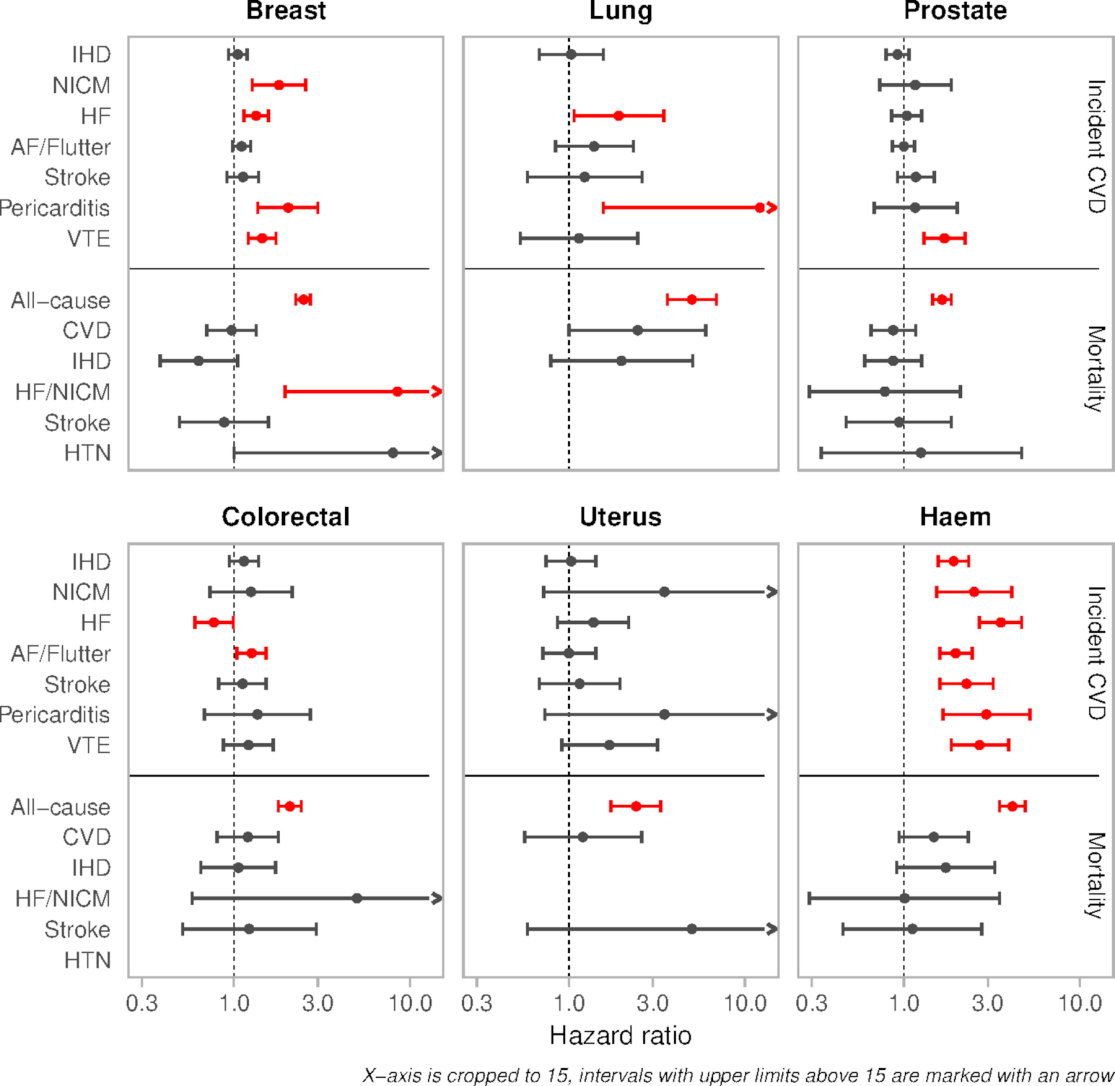
Associations of cancer exposure with incident cardiovascular disease and mortality outcomes. Results are association of cancer exposure with incident outcomes presented as subdistribution HRs and 95% CIs from competing risk regression, except for all-cause death where we report HR from Cox hazard proportional regression. HRs and 95% CIs are presented on a log_10_ scale. The comparators are propensity matched non-cancer controls. The dots represent the point estimate, and the intervals are the CIs. The greyed-out intervals indicate statistically non-significant associations. AF, atrial fibrillation; CVD, cardiovascular disease; NICM, non-ischaemic cardiomyopathies; Haem, haematological; HF, heart failure; HTN, hypertension; IHD, ischaemic heart disease.

Compared with matched non-cancer controls, those with past breast cancer had over twofold greater risk of incident pericarditis (SHR 2.03 (1.36, 3.00); p=0.0004), 80% greater risk of incident NICM (SHR 1.80 (1.27, 2.56), p=0.0008), and 45% greater risk of incident VTE (SHR 1.45 (1.21, 1.73); p=6.61×10^−5^) (table 3, figure 1). Breast cancer history was associated with 8.5-fold greater risk of death from HF or NICM (SHR 8.50 (1.95, 36.97); p=0.004) and eightfold greater risk of death from hypertensive diseases (SHR 8.00 (1.00, 64.07); p=0.05).

**Table 3:** Associations of cancer patients with incident cardiovascular events compared with propensity matched non-cancer controls

	Breast	Lung	Prostate	Colorectal	Uterus	Haematological
Incident disease						
IHD	1.05 (0.93, 1.19)	1.03 (0.68, 1.57)	0.92 (0.79, 1.07)	1.14 (0.94, 1.38)	1.03 (0.74, 1.42)	**1.92 (1.57, 2.34**)
	0.428	0.899	0.297	0.181	0.868	2.02×10^−10^
NICM	**1.80 (1.27, 2.56**)	–	1.16 (0.73, 1.86)	1.25 (0.73, 2.14)	3.49 (0.72, 16.78)	**2.51 (1.54, 4.10**)
	0.0008	–	0.543	0.416	0.121	0.002
Heart failure	**1.34 (1.14, 1.57**)	**1.92 (1.07, 3.46**)	1.04 (0.85, 1.26)	**0.77 (0.60, 0.99**)	1.38 (0.86, 2.18)	**3.56 (2.69, 4.66**)
	0.0004	0.029	0.72	0.044	0.181	1.19×10^−19^
AF/flutter	1.11 (0.98, 1.25)	1.39 (0.84, 2.32)	1.00 (0.86, 1.15)	**1.26 (1.04, 1.52**)	1.00 (0.71, 1.42)	**1.97 (1.60, 3.22**)
	0.114	0.206	0.969	0.02	0.996	4.43×10^−6^
Stroke	1.13 (0.91, 1.38)	1.23 (0.58, 2.61)	1.17 (0.92. 1.49)	1.12 (0.82, 1.52)	1.15 (0.68, 1.95)	**2.27 (1.60, 2.44**)
	0.259	0.575	0.194	0.48	0.59	2.62×10^−10^
Pericarditis	**2.03 (1.36, 3.00**)	**12.18 (1.57, 94.63**)	1.16 (0.68, 2.01)	1.36 (0.68, 2.72)	3.49 (0.73, 16.95)	**2.94 (1.67, 5.21**)
	0.0004	0.017	0.585	0.385	0.119	0.0002
VTE	**1.45 (1.21, 1.73**)	1.14 (0.53, 2.46)	**1.70 (1.30, 2.23**)	1.21 (0.87, 1.67)	1.70 (0.91, 3.19)	**2.69 (1.86, 3.94**)
	6.61×10^-5^	0.736	0.0001	0.2639	0.095	2.47×10^−7^
Mortality outcomes						
All-cause	**2.48 (2.25, 2.72**)	**5.00 (3.63, 6.89**)	**1.65 (1.46, 1.86**)	**2.08 (1.79, 2.41**)	**2.41 (1.73, 3.32**)	**4.14 (3.49, 4.90**)
	3.65×10^–80^	7.25×10^-21^	2.40×10^–16^	1.30×10^–21^	3.06×10^-7^	3.10×10^−59^
Any CVD	0.97 (0.70, 1.34)	2.46 (1.00, 5.99)	0.87 (0.65, 1.17)	1.20 (0.80, 1.79)	1.20 (0.56, 2.59)	1.48 (0.94, 2.32)
	0.871	0.05	0.371	0.374	0.64	0.087
IHD	0.63 (0.38, 1.05)	1.99 (0.79, 5.05)	0.87 (0.60, 1.26)	1.06 (0.65, 1.72)	–	1.73 (0.91, 3.29)
	0.079	0.14	0.461	0.820	–	0.090
Heart failure or NICM	**8.50 (1.95, 36.97**)	–	0.78 (0.29, 2.10)	5.00 (0.58, 42.95)	–	1.01 (0.29, 3.49)
	0.004	–	0.615	0.142	–	0.991
Stroke	0.88 (0.49, 1.57)	–	0.94 (0.47, 1.86)	1.22 (0.51, 2.94)	5.00 (0.58, 42.95)	1.12 (0.45, 2.77)
	0.656	–	0.853	0.652	0.142	0.806
Hypertensive diseases	**8.00 (1.00, 64.07**)	–	1.25 (0.34, 4.66)	–	–	–
	0.050	–	0.741	–	–	–

Results are subdistribution HR (95% CI) and p value associated with cancer exposure (vs no cancer). Blank cells indicate that no analysis was performed due to small number of outcomes (<5) in that category. Comparators are matched on age, sex, ethnicity, deprivation, education, blood pressure, heart rate, body mass index, glycated haemoglobin, random glucose, total cholesterol, high density lipoprotein, low density lipoprotein, triglyceride level, physical activity, smoking, diabetes, hypertension and high cholesterol. The bold cells represent statistically significant associations.

AF, atrial fibrillation; CVD, cardiovascular disease; IHD, ischaemic heart disease; NICM, non-ischaemic cardiomyopathies; VTE, venous thromboembolism.

### Lung cancer

Among the cancer sites considered, participants with a history of lung cancer (n=313) had the highest rates of incident CVD (49.4%), all-cause death (51.1%), and CVD death (5.4%). The most common incident CVDs were IHD (12.8%), AF/flutter (12.1%) and HF (10.2%). Among participants with lung cancer who died, 10.1% (17/160) died of a primary cardiovascular cause.

Lung cancer was associated with over 12-fold greater risk of incident pericarditis (SHR 12.18 (1.57, 94.63); p=0.017), 88% greater risk of incident HF (SHR 1.88 (1.07, 3.29); p=0.029), and almost 2.5-fold greater risk of CVD death (SHR 2.46 (1.00, 5.99); p=0.05). The risk of IHD death was increased in lung cancer patients, although with wide CIs (SHR 1.99 (0.79, 5.05); p=0.14).

### Prostate cancer

Among 3291 participants with prostate cancer, 40.6% developed incident CVD and 20.8% died. Primary cardiovascular deaths contributed 12.2% (83/683) of all deaths. The most common incident CVDs were IHD (11.7%), AF/flutter (11.6%), and HF (6.2%). Incident stroke and VTE each occurred in 4.5%, NICMs in 1.2% and pericarditis in 0.9%.

Compared with matched non-cancer controls, participants with prostate cancer had increased risk of incident VTE (SHR 1.70 (1.30, 2.23); p=0.0001) and all-cause death (HR 1.65 (1.46, 1.86); p=2.40×10^−16^). Associations with all other outcomes were statistically non-significant.

### Colorectal cancer

One-third (803/2412) of participants with colorectal cancer developed incident CVD, 20.7% died and 2.2% died of primary cardiovascular causes (10.8% of all deaths: 54/499). The most common incident CVDs were IHD (10.2%), AF/flutter (9.8%), and HF (4.4%).

Participants with colorectal cancer had 26% greater risk of incident AF/flutter (SHR 1.26 (1.04, 1.52); p=0.02) compared with matched non-cancer controls. Colorectal cancer was associated with higher risk of HF/NICM death, but with wide CIs (SHR 5.00 (0.58, 42.95); p=0.14). Aside from all-cause death, there was no statistically significant difference in risk of any other outcome.

### Uterine cancer

Among the 937 participants with uterine cancer, 26.7% developed incident CVD and 12.1% died. Primary cardiovascular deaths contributed 10.6% (12/113) of all deaths. The most common incident CVDs were AF/flutter (7.4%), IHD (7.3%) and HF (4.5%). Incident stroke occurred in 3.2%, VTE in 2.9% and NICMs and pericarditis were each observed in 0.7% of individuals.

Compared with matched non-cancer controls, uterine cancer patients had increased (statistically non-significant) risk of incident NICM (SHR 3.49 (0.72, 16.78); p=0.12), pericarditis (SHR 3.49 (0.73, 16.95); p=0.12) and stroke death (SHR 5.00 (0.58, 42.95); p=0.14).

### Haematological cancer

Among 2230 participants with past haematological cancer, 48.4% (n=1080) developed incident CVD and 27.1% died. A total of 7.8% (47/605) of all deaths were attributed to a primary cardiovascular cause. The most common CVDs were IHD (12.8%), AF/flutter (11.6%), and HF (10.2%). Incident stroke and VTE each occurred in 4.6%, NICMs in 2.6% and pericarditis in 2.1% of haematological cancer patients.

Participants with past haematological cancer had significantly greater risk of all incident CVDs (table 3, figure 1). The risk of incident HF was increased by over 3.5-fold (SHR 3.56 (2.69, 4.66); p=1.19×10^−19^), pericarditis by almost threefold (SHR 2.94 (1.67, 5.21); p=0.0002)], and there was over 2.5-fold greater risk of both incident VTE (SHR 2.69 (1.86, 3.94); p=2.47×10^−7^] and NICM (SHR 2.51 (1.54, 4.10); p=0.002). There was almost twofold increased risk of incident AF/flutter (SHR 1.97 (1.60, 2.44); p=2.62×10^−10^) and IHD (SHR 1.92 (1.57, 2.34); p=2.02×10^−10^). Associations with CVD mortality outcomes were statistically non-significant; however, participants with a history of haematological cancer appeared at higher risk of CVD (SHR 1.48 (0.94, 2.32); p=0.087) and IHD (SHR 1.73 (0.91, 3.29); p=0.090) death.

Associations with incident events were broadly similar across myeloma, leukaemia, and lymphomas (online supplemental tables 5 and 6).

### Sensitivity analyses

In analyses limiting to cases with complete data, associations remained similar across all outcomes (online supplemental tables 7 and 8). The results were consistent in cause-specific Cox regression models (online supplemental table 9) and when restricting to participants diagnosed with cancer within 5 years of baseline (online supplemental tables 10 and 11). The interaction of cancer exposure with time from diagnosis was non-significant for all models, except for the association of lung cancer with incident stroke, where risk was higher in the earlier years after cancer incidence.

### Associations with CMR metrics

**Figure 2:**
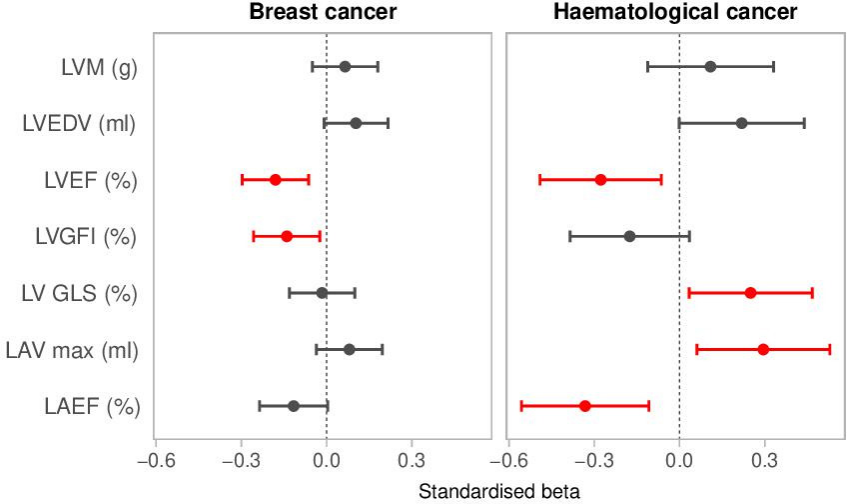
Association of breast and haematological cancer exposure with CMR metrics. Results are standardised beta-coefficients and 95% CIs, thus representing SD change in CMR metrics with change in cancer exposure status from non-cancer to cancer. CMR, cardiovascular magnetic resonance; GLS, LV global longitudinal strain; LA, left atrium; LAEF, LA ejection fraction; LAV, LA maximum volume; LV, left ventricle; LVEDV, LV end-diastolic volume; LVEF, LV ejection fraction; LVGFI, LV global function index; LVM, LV mass.

We investigated associations of past cancer with cardiovascular phenotypes in 1354 participants who had CMR data available (online supplemental table 12). Compared with matched non-cancer controls, participants with past haematological cancer had larger LVEDV, poorer LV function by both LVEF and LV GLS, larger LAV, and lower LAEF (table 4, figure 2). Breast cancer was associated with significantly poorer LV function by LVEF and LVGFI. These relationships were similar in individuals without CVD at imaging (online supplemental table 13).

**Table 4:** Association of cancer with CMR metrics

	Breast	Lung	Prostate	Colorectal*	Uterus*	Haem*
LVM (g)	0.07 (−0.05, 0.18)	−0.41 (−1.27, 0.46)	−0.01 (−0.14, 0.12)	−0.23 (−0.78, 0.32)	0.14 (−0.23, 0.51)	0.11 (−0.11, 0.33)
	0.27	0.33	0.84	0.40	0.45	0.33
LVEDV (mL)	0.10 (−0.01, 0.22)	−0.56 (−1.48, 0.35)	0.05 (−0.08, 0.18)	−0.32 (−0.85, 0.22)	−0.01 (−0.37, 0.35)	0.22 (−0.00, 0.44)
	0.07	0.21	0.44	0.24	0.94	0.05
LVEF (%)	−**0.18 (−0.30, −0.06**)	0.62 (−0.26, 1.50)	0.02 (−0.10, 0.15)	−0.12 (−0.60, 0.36)	0.03 (−0.34, 0.41)	−**0.28 (−0.49, −0.06**)
	0.003	0.15	0.73	0.61	0.87	0.01
LVGFI (%)	−**0.14 (−0.26, −0.02**)	0.25 (−0.68, 1.18)	0.05 (−0.07, 0.18)	−0.13 (−0.59, 0.34)	−0.06 (−0.45, 0.33)	−0.18 (−0.39, 0.04)
	0.02	0.56	0.41	0.58	0.76	0.10
LV GLS (%)	−0.02 (−0.13, 0.10)	−0.87 (−1.71, −0.04)	−0.03 (−0.17, 0.11)	0.38 (−0.26, 1.02)	0.33 (−0.14, 0.80)	**0.25 (0.03, 0.47**)
	0.78	0.05	0.65	0.24	0.17	0.02
LAV max (mL)	0.08 (−0.04, 0.20)	−0.82 (−1.69, 0.05)	0.02 (−0.11, 0.16)	−0.35 (−0.76, 0.05)	−0.01 (−0.39, 0.37)	**0.30 (0.06, 0.53**)
	0.18	0.06	0.75	0.09	0.96	0.01
LAEF (%)	−0.12 (−0.24, 0.00)	0.42 (−0.11, 0.94)	−0.02 (−0.15, 0.11)	0.15 (−0.24, 0.54)	−0.07 (−0.41, 0.27)	−**0.33 (−0.56, −0.11**)
	0.06	0.11	0.74	0.45	0.68	0.004

The results are standardised beta-coefficients and 95% CIs, thus representing SD change in CMR metrics with change in cancer exposure status from non-cancer to cancer; for SD of each metric, please refer to online supplemental table 5. The bold and yellow shaded cells represent statistically significant associations.

*Doubly robust model.

GLS, LV global longitudinal strain; LA, left atrium; LAEF, LA ejection fraction; LAV, LA maximum volume; LV, left ventricle; LVEDV, LV end-diastolic volume; LVEF, LV ejection fraction; LVGFI, LV global function index; LVM, LV mass.

## Discussion

### Summary of findings

In this large population-based study, covering an average of 12 years prospective follow-up, past cancer was linked to increased risk of a wide range of incident cardiovascular outcomes and adverse remodelling, independent of shared vascular risk factors. Previous haematological cancer was linked to increased incidence of all CVDs considered, poorer LV function (by LVEF and GLS), larger LV and LA size, and poorer LA function (lower LAEF). Past breast cancer was linked to increased incidence of NICM, HF, pericarditis, VTE, HF/NICM mortality, hypertensive disease death, and poorer LV function (by LVEF and LVGFI). Lung cancer was associated with increased risk of incident HF, pericarditis and CVD death. Colorectal cancer was associated with increased risk of incident AF/flutter. Prostate cancer was linked to increased VTE risk.

### Comparison with previous work

The most common incident CVDs in our cancer-exposed cohort were IHD, AF/flutter, and HF. This distribution reflects both the risk factor profile of individuals with cancer and general population trends.^14^ Consistent with previous reports, we found high burden of vascular risk factors in participants with cancer.^15 16^ The observed CVD patterns are similar to studies from China and the USA.^15 17^ In our cancer cohort, 8.2% of deaths were attributed to primary cardiovascular causes. Similarly, an analysis of the UK Clinical Primary Records Datalink identified CVD as the primary cause of death in 9.7% of men and 7.7% of women with cancer.^18^

Our work extends previous reports by isolating cardiovascular risk associated with cancer independent of shared risk factors. A recent study from the UK used linked primary care and hospitalisation records to examine risk of incident disease-specific CVDs in patients with cancer independent of vascular risk factors.^3^ Our findings validate these observations in an independent cohort and provide new insights by considering disease associations alongside CMR remodelling.

Participants with previous haematological cancer had significantly increased risk of all incident CVDs. They also had increased size and poorer function of both the LA and LV. Haematological cancer patients are exposed to many cardiotoxic cancer therapies such as tyrosine kinase inhibitors,^19^ cyclophosphamide,^20^ anthracyclines,^21^ and mediastinal radiotherapy.^22^ The observed pattern of LV remodelling associated with haematological cancer may reflect subclinical cardiotoxicity, indicating a dilated LV with lower ejection fraction and poorer longitudinal function, and is consistent with our finding of increased risk of incident NICM and HF. The atrial remodelling patterns of a dilated and poorly functioning LA may reflect haemodynamic consequences of increased LV filling pressures that accompanies HF. There may also be direct effects on the atria via radiotherapy or other treatments. Regardless of underlying mechanism, atrial remodelling is both precipitated by and predisposes to AF, which we found to be significantly associated with haematological cancer history. We also found increased risk of stroke associated with past haematological cancer, which is likely driven by both ischaemic and haemorrhagic mechanisms, with the latter precipitated by coagulopathies related to the primary cancer and greater use of anticoagulants in these patients.

Increased risk of VTE was observed in participants with haematological, breast, and prostate cancer. Many factors promote a prothrombotic state in the setting of cancer, such as the systemic biological processes of the cancer itself, tumour compression effects, chemotherapy, and long-term indwelling venous catheters. Previous studies have documented augmented risk of VTE in patients with cancer.^23^ In our study, the magnitude of increased VTE risk was highest among participants with past haematological cancer.

Radiation-induced heart disease has a range of possible manifestations.^24^ Mediastinal radiotherapy has been linked to initiation and progression of atherosclerosis. Patients with lymphomas are often exposed to mediastinal radiotherapy, which may be a driver of the increased risk of IHD in participants with previous haematological cancer in our cohort. Our findings are consistent with a previous study by van Nimwegen *et al*,^25^ who also report increased risk of IHD in Hodgkin lymphoma survivors and attribute this, in part, to radiotherapy exposure.

Participants with previous lung, breast or haematological cancer had increased risk of pericardial disease, with lung cancer patients having a markedly increased risk (over 12-fold). This may reflect metastatic disease presentations. Pericardial disease may also be an adverse consequence of mediastinal radiotherapy,^24^ which is common in all three cancers.

Participants with breast cancer had increased risk of incident HF, incident NICMs and death from HF or NICM. Furthermore, breast cancer history was associated with poorer LV function by LVGFI and LVEF. These observations likely reflect cardiotoxicity linked to breast cancer therapies.^21 26^ An interesting observation in our results was a markedly increased risk of death due to hypertensive disease (eightfold increase) in participants with previous breast cancer, which may reflect suboptimal control of hypertension in this cohort.

Participants with uterine cancer had the highest average body mass index of all cancers, high rates of hypertension and diabetes and increased risk of stroke death. The clustering of cardiometabolic factors has been previously reported in uterine cancer.^27 28^ In our analysis, uterine cancer was linked to increased stroke mortality but with very wide CIs.

### Clinical implications

Patients with cancer have a constellation of demographic and clinical risk factors that place them at higher cardiovascular risk. Our findings underscore the importance of controlling modifiable risk factors for all patients during and after their cancer treatment, as well as specific areas of risk where surveillance and/or preventive strategies should be focused. Importantly, we demonstrate that past cancer confers an increased risk of cardiovascular events, independent of traditional vascular risk factors and that this risk may extend several years beyond the initial cancer diagnosis. Thus, our results support consideration of cancer-specific exposures in cardiovascular risk stratification and lower thresholds for treatment of modifiable risk factors in this patient group. We demonstrate particular vulnerability of individuals with past breast and haematological cancer, who appeared at greatest risk, both with regards risk of incident clinical disease and adverse cardiac remodelling.

We found significant associations between breast and haematological cancer history and selected CMR metrics, even in the absence of prevalent CVD. The most consistent associations were observed with LVEF. We also demonstrate potential value of LVGFI, GLS, and LAEF as emerging novel imaging biomarkers of subclinical disease.

### Limitations

Ascertainment of incident outcomes from health records may be subject to miscoding. We may be underpowered to detect associations in cancers with small sample sizes (eg, lung and uterine). Our dataset does not permit characterisation by cancer histology or stage. Information about specific cancer therapies was not available, and we cannot make inferences about treatment-specific effects. We are unable to consider ethnic disparities as our sample comprises a predominantly white cohort; future studies in more diverse cohorts are needed.

## Conclusions

Individuals with past cancer have heightened cardiovascular risk, which appears independent of vascular risk factors and persists several years after initial cancer diagnosis. The pattern of CVDs varies by cancer site, likely reflecting specific characteristics of the cancer and its therapies. CMR measures of LV and LA structure and function provide preclinical indicators of cardiovascular health in this context.

## Data Availability

Data may be obtained from a third party and are not publicly available. This research was conducted using the UK Biobank resource under access application 2964. UK Biobank will make the data available to all bona fide researchers for all types of health-related research that is in the public interest, without preferential or exclusive access for any persons. All researchers will be subject to the same application process and approval criteria as specified by UK Biobank. For more details on the access procedure, see the UK Biobank website: http://www.ukbiobank.ac.uk/register-apply.
