# Supplementary material for: Incident cardiovascular events and imaging phenotypes in UK Biobank participants with past cancer
Source: Heart. 2023 Apr 18;109(13):1007–15. doi: 10.1136/heartjnl-2022-321888 (PMC10314020; doi:10.1136/heartjnl-2022-321888)
Supplement: Supplementary data [file heartjnl-2022-321888supp001.pdf]

Supplementary Figure 1. Postulated causal pathways and potential and true confounders of the relationship between cancer and cardiovascular health

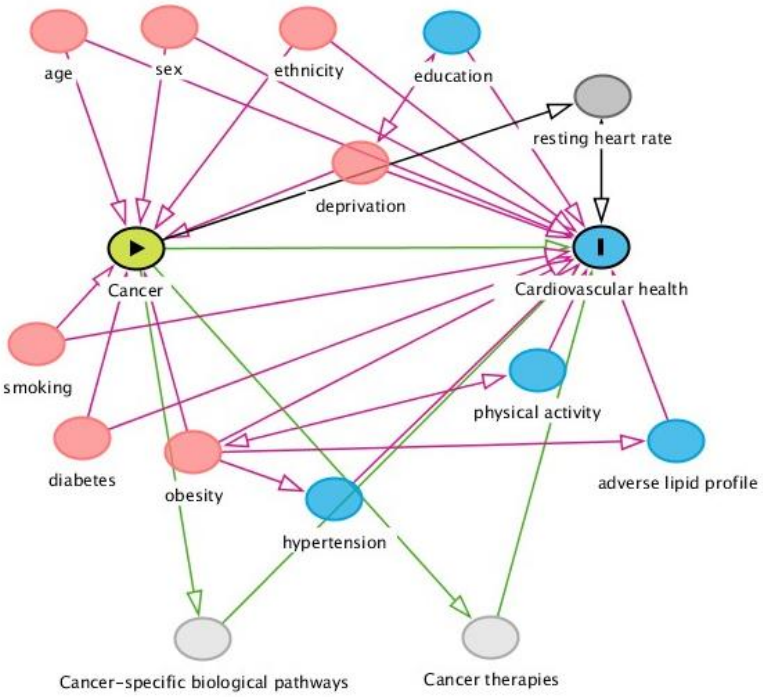

Supplementary Figure 1 footnote. Figure created using the dagitty package: Johannes Textor, Benito van der Zander, Mark K. Gilthorpe, Maciej Liskiewicz, George T.H. Ellison. Robust causal inference using directed acyclic graphs: the R package 'dagitty'. *International Journal of Epidemiology* 45(6):1887-1894, 2016.

Supplementary Figure 2. Balance plots for propensity score matching in the baseline set

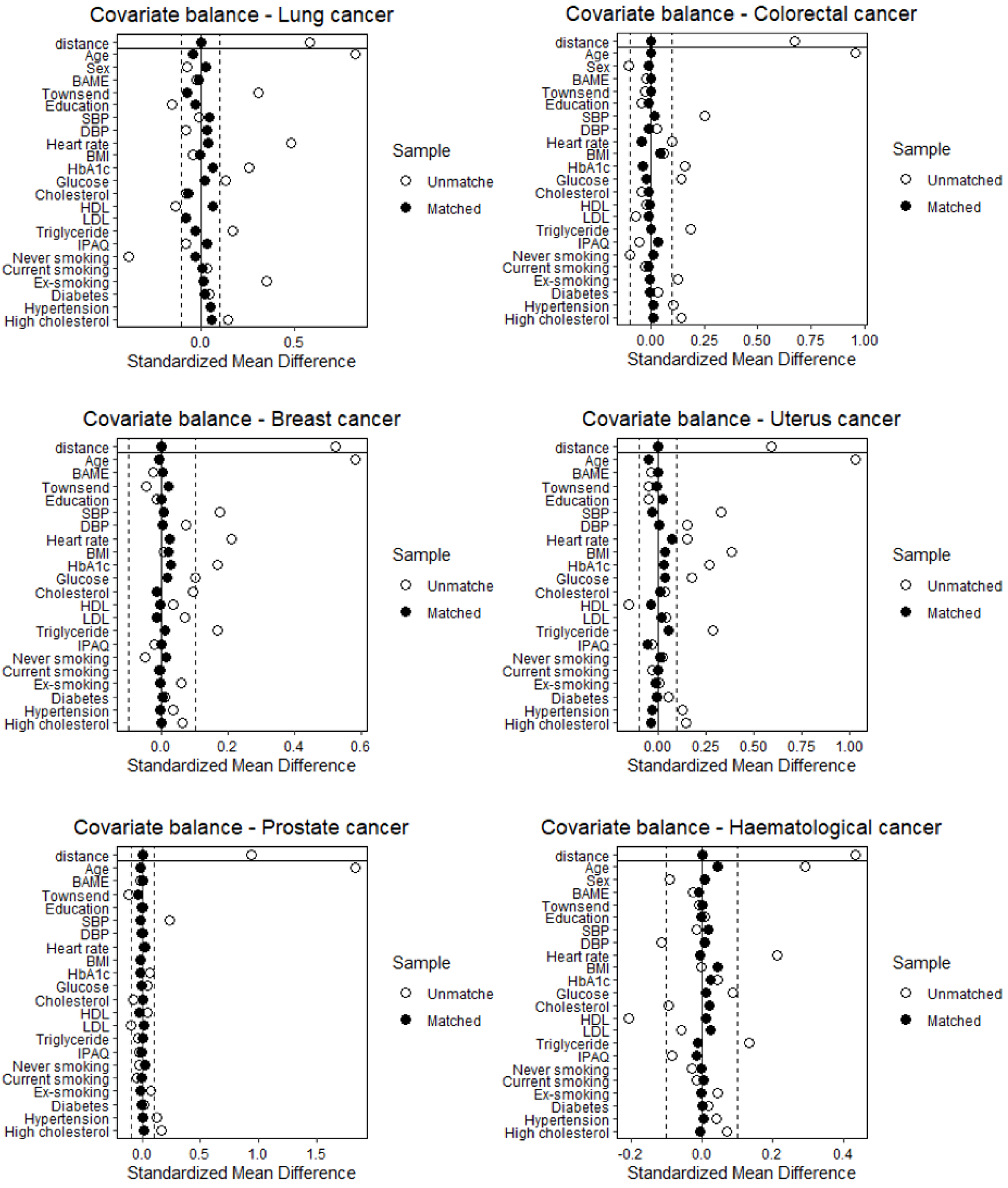

Supplementary Figure 2 footnote. Vertical dashed lines show threshold of 0.1 standardised mean difference. There was good balance of overall propensity score and individual covariates for all cancer categories in the baseline set. BMI: body mass index; DBP: diastolic blood pressure; HbA1c: glycated

haemoglobin, HDL: high density lipoprotein, IPAQ: international physical activity questionnaire; LDL: low density lipoprotein; METS: metabolic equivalent; SBP: systolic blood pressure.

Supplementary Figure 3. Balance plots for propensity score matching in the imaging set

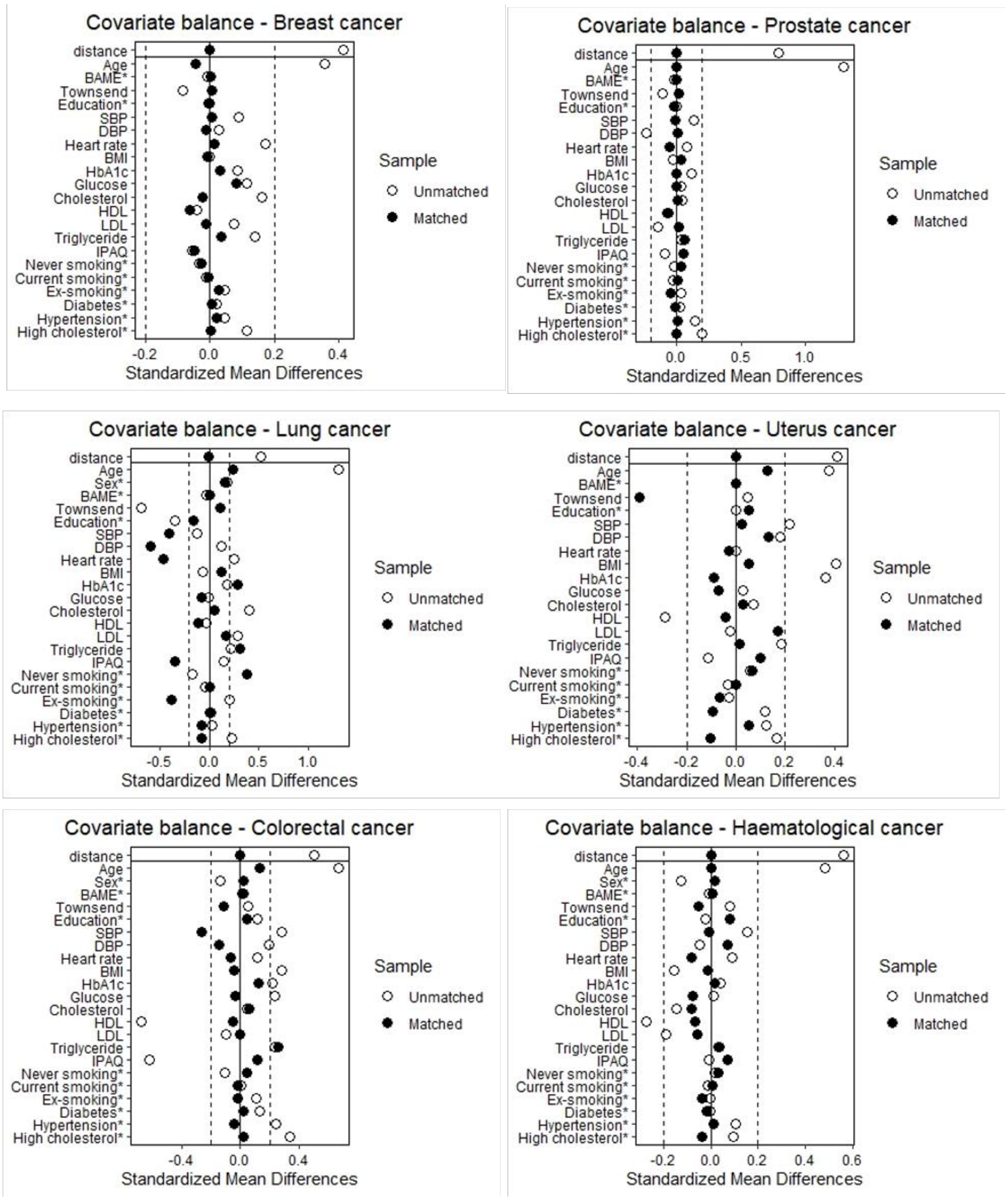

Supplementary Figure 3 footnote. Vertical dashed lines show threshold of 0.1 standardised mean difference. Dotted lines show caliper threshold of 0.2 standard deviations. We excluded 5 men with breast cancer. In the lung cancer category, age, sex, smoking, education, SBP, DBP, TG, IPAQ, heart rate and hba1c all have SMD >0.2. Townsend, BMI, HDL, LDL, hypertension and high cholesterol>0.1. For prostate cancer, 1 pair outside caliper was discarded. For colorectal cancer, 1 pair outside caliper discarded and age, ethnicity, Townsend, Education, SBP, DBP, hba1c,IPAQ, smoking

all had SMD >0.1. For uterine cancer, 1 pair was unmatched and Townsend score SMD>0.2, whilst age, qualifications,DBP,LDL,IPAQ, smoking, diabetes, hypertension, high cholesterol all had SMD >0.1. For haematological cancer, Education had SMD>0.1. In conclusion, covariate balance is good for breast and prostate cancer. Overall propensity is balanced for the outcomes, but some individual covariates lack balance (SMD>0.1) and thus we used a doubly robust approach by including these as covariates in the final models as per Nguyen et al. (Nguyen TL, Collins GS, Spence J, Daurès JP, Devereaux PJ, Landais P, Le Manach Y. Double-adjustment in propensity score matching analysis: choosing a threshold for considering residual imbalance. BMC Med Res Methodol. 2017 Apr 28;17(1):78. doi: 10.1186/s12874-017-0338-0.). BMI: body mass index; DBP: diastolic blood pressure; HbA1c: glycated haemoglobin, HDL: high density lipoprotein, IPAQ: international physical activity questionnaire; LDL: low density lipoprotein; METS: metabolic equivalent; SBP: systolic blood pressure.

Supplementary Figure 4. Flow of participants included in study

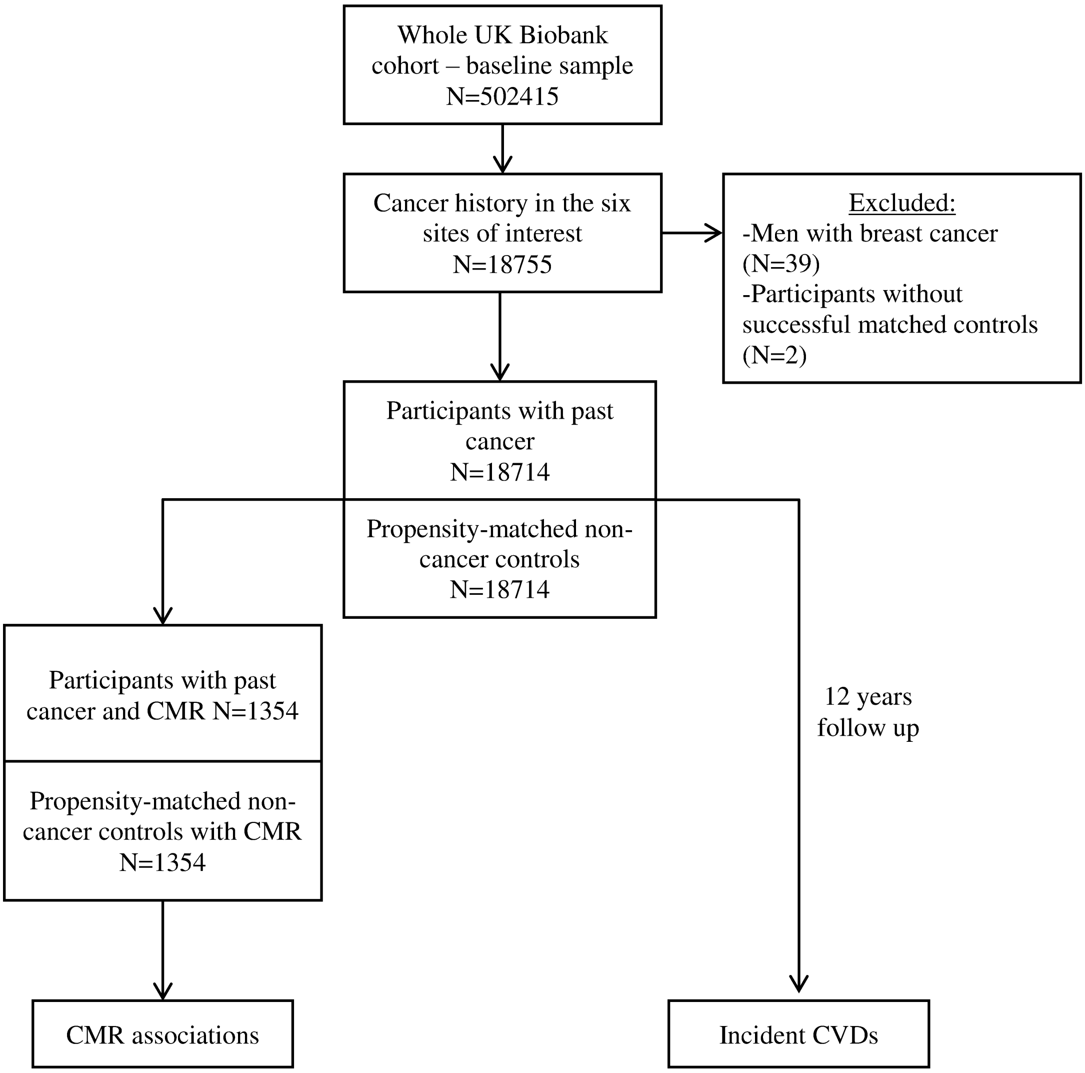

Supplementary Figure 4 footnote. CVD: cardiovascular disease.

Supplementary Table 1. ICD-9 and ICD-10 codes used for ascertainment of cancer status

Cancer site	ICD9/10 code	Description
Breast	1740	Malignant neoplasm of female breast - nipple and areola
	1743	Malignant neoplasm of female breast - lower-inner quadrant
	1744	Malignant neoplasm of female breast - upper-outer quadrant
	1745	Malignant neoplasm of female breast - lower-outer quadrant
	1748	Malignant neoplasm of female breast - other site
	1749	Malignant neoplasm of female breast - unspecified site
	1740	Malignant neoplasm of female breast - nipple and areola
	1743	Malignant neoplasm of female breast - lower-inner quadrant
	C50.0	Nipple and areola
	C50.1	Central portion of breast
	C50.2	Upper-inner quadrant of breast
	C50.3	Lower-inner quadrant of breast
	C50.4	Upper-outer quadrant of breast
	C50.5	Lower-outer quadrant of breast
	C50.6	Axillary tail of breast
	C50.8	Overlapping lesion of breast
	C50.9	Breast, unspecified
Lung	1623	Malignant neoplasm of upper lobe, bronchus or lung
	1629	Malignant neoplasm of bronchus and lung, unspecified
	C34.0	Main bronchus
	C34.1	Upper lobe, bronchus, or lung
	C34.2	Middle lobe, bronchus, or lung
	C34.3	Lower lobe, bronchus, or lung
	C34.8	Overlapping lesion of bronchus and lung
	C34.9	Bronchus or lung, unspecified
Prostate	1859	Malignant neoplasm of prostate
	C61	Malignant neoplasm of prostate
Colorectal	1530	Malignant neoplasm of colon, hepatic flexure
	1532	Malignant neoplasm of descending colon
	1533	Malignant neoplasm of sigmoid colon
	1534	Malignant neoplasm of caecum
	1536	Malignant neoplasm of ascending colon
	1537	Malignant neoplasm of colon, splenic flexure
	1539	Malignant neoplasm of colon, unspecified
	C18.0	Caecum
	C18.1	Appendix
	C18.2	Ascending colon
	C18.3	Hepatic flexure
	C18.4	Transverse colon
	C18.5	Splenic flexure
	C18.6	Descending colon
	C18.7	Sigmoid colon
	C18.8	Overlapping lesion of colon
	C18.9	Colon, unspecified
	C19	Malignant neoplasm of rectosigmoid junction
	C20	Malignant neoplasm of rectum
Uterus	1820	Malignant neoplasm of corpus uteri, except isthmus
	C54.0	Isthmus uteri
	C54.1	Endometrium
	C54.2	Myometrium
	C54.3	Fundus uteri
	C54.8	Overlapping lesion of corpus uteri
	C54.9	Corpus uteri, unspecified
	C55	Malignant neoplasm of uterus, part unspecified
Haematological	2001	Lymphosarcoma
	2015	Hodgkin's disease, nodular sclerosis
	2016	Hodgkin's disease, mixed cellularity

Cancer site	ICD9/10 code	Description
	2017	Hodgkin's disease, lymphocytic depletion
	2019	Hodgkin's disease, unspecified
	2020	Nodular lymphoma
	2024	Leukaemic reticuloendotheliosis
	2028	Other lymphomas
	2029	Other malig. neoplasm of lymphoid and histiocytic tissue
	2040	Acute lymphoid leukaemia
	2050	Acute myeloid leukaemia
	2051	Chronic myeloid leukaemia
	2059	Unspecified myeloid leukaemia
	C81.0	Lymphocytic predominance
	C81.1	Nodular sclerosis
	C81.2	Mixed cellularity
	C81.3	Lymphocytic depletion
	C81.4	Lymphocyte-rich classical Hodgkin lymphoma
	C81.7	Other Hodgkin's disease
	C81.9	Hodgkin's disease, unspecified
	C82.0	Small cleaved cell, follicular
	C82.1	Mixed small cleaved and large cell, follicular
	C82.2	Large cell, follicular
	C82.3	Follicular lymphoma grade IIIa
	C82.4	Follicular lymphoma grade IIIb
	C82.5	Diffuse follicle centre lymphoma
	C82.6	Cutaneous follicle centre lymphoma
	C82.7	Other types of follicular non-Hodgkin's lymphoma
	C82.9	Follicular non-Hodgkin's lymphoma, unspecified
	C83.0	Small cell (diffuse)
	C83.1	Small cleaved cell (diffuse)
	C83.2	Mixed small and large cell (diffuse)
	C83.3	Large cell (diffuse)
	C83.4	Immunoblastic (diffuse)
	C83.5	Lymphoblastic (diffuse)
	C83.6	Undifferentiated (diffuse)
	C83.7	Burkitt's tumour
	C83.8	Other types of diffuse non-Hodgkin's lymphoma
	C83.9	Diffuse non-Hodgkin's lymphoma, unspecified
	C84.0	Mycosis fungoides
	C84.1	Sezary's diease
	C84.3	Lymphoepithelioid lymphoma
	C84.4	Peripheral T-cell lymphoma
	C84.5	Other and unspecified T-cell lymphomas
	C84.6	Anaplastic large cell lymphoma, ALK-positive
	C84.7	Anaplastic large cell lymphoma, ALK-negative
	C84.8	Cutaneous T-cell lymphoma, unspecified
	C84.9	Mature T/NK-cell lymphoma, unspecified
	C85.0	Lymphosarcoma
	C85.1	B-cell lymphoma, unspecified
	C85.2	Mediastinal (thymic) large B-cell lymphoma
	C85.7	Other specified types of non-Hodgkin's lymphoma
	C85.9	Non-Hodgkin's lymphoma, unspecified type
	C86.0	Extranodal NK/T-cell lymphoma, nasal type
	C86.2	Enteropathy-type (intestinal) T-cell lymphoma
	C86.3	Subcutaneous panniculitis-like T-cell lymphoma
	C86.4	Blastic NK-cell lymphoma
	C86.5	Angioimmunoblastic T-cell lymphoma
	C86.6	Primary cutaneous CD30-positive T-cell proliferations
	C88.0	Waldenstrom's macroglobulinaemia
	C88.2	Gamma heavy chain disease
	C88.3	Immunoproliferative small intestinal disease

Cancer site	ICD9/10 code	Description
	C88.4	Extranodal marginal zone B-cell lymphoma of mucosa-associated lymphoid tissue [MALT-lymphoma]
	C88.7	Other malignant immunoproliferative diseases
	C88.9	Malignant immunoproliferative disease, unspecified
	C90.0	Multiple myeloma
	C90.1	Plasma cell leukaemia
	C90.2	Plasmacytoma, extramedullary
	C90.3	Solitary plasmacytoma
	C91.0	Acute lymphoblastic leukaemia
	C91.1	Chronic lymphocytic leukaemia
	C91.2	Subacute lymphocytic leukaemia
	C91.3	Prolymphocytic leukaemia
	C91.4	Hairy-cell leukaemia
	C91.5	Adult T-cell leukaemia
	C91.6	Prolymphocytic leukaemia of T-cell type
	C91.7	Other lymphoid leukaemia
	C91.8	Mature B-cell leukaemia Burkitt-type
	C92.0	Acute myeloid leukaemia
	C92.1	Chronic myeloid leukaemia
	C92.2	Subacute myeloid leukaemia
	C92.3	Myeloid sarcoma
	C92.4	Acute promyelocytic leukaemia
	C92.5	Acute myelomonocytic leukaemia
	C92.6	Acute myeloid leukaemia with 11q23-abnormality
	C92.7	Other myeloid leukaemia
	C92.8	Acute myeloid leukaemia with multilineage dysplasia
	C93.0	Acute monocytic leukaemia
	C93.1	Chronic monocytic leukaemia
	C93.3	Juvenile myelomonocytic leukaemia
	C93.9	Monocytic leukaemia, unspecified
	C94.0	Acute erythraemia and erythroleukaemia
	C94.2	Acute megakaryoblastic leukaemia
	C94.4	Acute panmyelosis
	C94.5	Acute myelofibrosis
	C94.6	Myelodysplastic and myeloproliferative disease, not elsewhere classified
	C94.7	Other specified leukaemias
	C95.0	Acute leukaemia of unspecified cell type
	C95.1	Chronic leukaemia of unspecified cell type
	C95.9	Leukaemia, unspecified
	C96.1	Malignant histiocytosis
	C96.2	Malignant mast cell tumour
	C96.3	True histiocytic lymphoma
	C96.4	Sarcoma of dendritic cells (accessory cells)
	C96.5	Multifocal and unisystemic Langerhans-cell histiocytosis
	C96.6	Unifocal Langerhans-cell histiocytosis
	C96.7	Other specified malignant neoplasms of lymphoid, haematopoietic and related tissue
	C96.8	Histiocytic sarcoma
	C96.9	Malignant neoplasms of lymphoid, haematopoietic and related tissue, unspecified

Supplementary Table 1 footnote. ICD: international classification of disease

Supplementary Table 2. Ascertainment of CVD outcomes, ICD and UK Biobank field codes

Source	ICD code/UKB filed	Description
**Ischaemic heart disease (IHD)**		
ICD9	4139	Angina pectoris
	4140	Coronary atherosclerosis
	4141	Aneurysm of heart
	4148	Other specified forms of chronic ischaemic heart disease
	4149	Chronic ischaemic heart disease, unspecified
	4119	Other acute and subacute forms of ischaemic heart disease
Self-report	20002	Angina
ICD10	I20	Angina pectoris
	I24	Other acute ischaemic heart diseases
	I25	Chronic ischaemic heart disease
First occurrences	131296	Angina pectoris
	131304	Other acute ischaemic heart diseases
	131306	Chronic ischaemic heart disease
Diagnosed by doctor	3627	Age angina diagnosed
	6150: 2	Angina
**Ischaemic heart disease (Myocardial infarction)**		
ICD9	4109	Acute myocardial infarction
	4129	Old myocardial infarction
Self-report	20002	Heart attack/myocardial infarction
ICD9	410	Acute myocardial infarction
	411	Other acute and subacute forms of ischaemic heart disease
	412	Old myocardial infarction
ICD10	I21	Acute myocardial infarction
	I22	Subsequent myocardial infarction
	I23	Certain current complications following acute myocardial infarction
First occurrences	131298	Acute myocardial infarction
	131300	Subsequent myocardial infarction
	131302	Certain current complications following acute myocardial infarction
Diagnosed by doctor	3894	Age heart attack diagnosed
	6150: 1	Heart attack
Algorithm	42000	Date of myocardial infarction
**Non-ischaemic cardiomyopathies**		
ICD9	4254	Other primary cardiomyopathies
Self-report	20002	Cardiomyopathy
	20002	Hypertrophic cardiomyopathy (HCM / HOCM)
ICD10	I42	Cardiomyopathy
	I43	Cardiomyopathy in diseases classified elsewhere
	I11	Hypertensive heart disease
	I13	Hypertensive heart and renal disease
First occurrences	131338	Cardiomyopathy
	131340	Cardiomyopathy in diseases classified elsewhere
	131288	Hypertensive heart disease
	131292	Hypertensive heart and renal disease
**Heart failure (unspecified aetiology)**		
ICD9	4280	Congestive heart failure
	4281	Left heart failure
Self-report	20002	Heart failure/pulmonary oedema
ICD10	I50.0	Congestive heart failure
	I50.1	Left ventricular failure
	I50.9	Heart failure, unspecified
First occurrences	131354	Heart failure
**Cardiac arrhythmia (Atrial fibrillation)**		
Self-report	20002	Atrial fibrillation
ICD9	4273	Atrial fibrillation and flutter
ICD10	I48.0	Paroxysmal atrial fibrillation
	I48.1	Persistent atrial fibrillation

**Source**	**ICD code/UKB filed**	**Description**
	I48.2	Chronic atrial fibrillation
	I48.9	Atrial fibrillation and atrial flutter, unspecified
**Stroke**		
Self-report	20002	Stroke
	20002	Ischaemic stroke
	20002	Brain haemorrhage
ICD9	431	Intracerebral haemorrhage
	4349	Occlusion of cerebral arteries, unspecified
ICD10	I64	Stroke, not specified as haemorrhage or infarction
	I63	Cerebral infarction
	I61	Intracerebral haemorrhage
	I62	Other nontraumatic intracranial haemorrhage
First occurrences	131368	Date I64 first reported (stroke, not specified as haemorrhage or infarction)
	131366	Cerebral infarction
	131362	Intracerebral haemorrhage
	131364	other nontraumatic intracranial haemorrhage
Diagnosed by doctor	4056	Age stroke diagnosed
	6150: 3	Stroke
Algorithm	42006	Date of stroke
	42008	Date of ischaemic stroke
	42010	Date of intracerebral haemorrhage
**Pericarditis**		
ICD10	I30.0	Acute nonspecific idiopathic pericarditis
	I30.1	Infective pericarditis
	I30.8	Other forms of acute pericarditis
	I30.9	Acute pericarditis, unspecified
	I31.0	Chronic adhesive pericarditis
	I31.1	Chronic constrictive pericarditis
	I31.2	Haemopericardium, not elsewhere classified
	I31.3	Pericardial effusion (noninflammatory)
	I31.8	Other specified diseases of pericardium
	I31.9	Disease of pericardium, unspecified
	I32.0	Pericarditis in bacterial diseases classified elsewhere
	I32.1	Pericarditis in other infectious and parasitic diseases classified elsewhere1
	I32.8	Pericarditis in other diseases classified elsewhere
**Venous thromboembolism (DVT/PE)**		
ICD9	4151	Pulmonary embolism
	4538	Embolism and thrombosis of other specified veins
ICD10	I26.0	Pulmonary embolism with mention of acute cor pulmonale
	I26.9	Pulmonary embolism without mention of acute cor pulmonale
	I801	Phlebitis and thrombophlebitis of femoral vein
	I802	Phlebitis and thrombophlebitis of other deep vessels of lower extremities
	I803	Phlebitis and thrombophlebitis of lower extremities, unspecified
	I82.8	Embolism and thrombosis of other specified veins
	I82.9	Embolism and thrombosis of unspecified vein
Self report	20002	pulmonary embolism +/- DVT
	20002	deep venous thrombosis (DVT)
**Hypertensive disease (for death certificate, main/underlying cause of death)**		
ICD10	I10	Essential (primary) hypertension
	I11.0	Hypertensive heart disease with (congestive) heart failure
	I11.9	Hypertensive heart disease without (congestive) heart failure
	I12.0	Hypertensive renal disease with renal failure
	I12.9	Hypertensive renal disease without renal failure
	I13.0	Hypertensive heart and renal disease with (congestive) heart failure
	I13.1	Hypertensive heart and renal disease with renal failure
	I13.2	Hypertensive heart and renal disease with both (congestive) heart failure and renal failure
	I13.9	Hypertensive heart and renal disease, unspecified

Source	ICD code/UKB filed	Description
ICD9	I15.0	Renovascular hypertension
	I15.1	Hypertension secondary to other renal disorders
	I15.2	Hypertension secondary to endocrine disorders
	I15.8	Other secondary hypertension
	4010	Essential hypertension, specified as malignant
	4011	Essential hypertension, specified as benign
	4019	Essential hypertension, not specified as malignant or benign
	4039	Hypertensive renal disease, not specified as malignant or benign

Supplementary Table 2 footnote. CVD: cardiovascular disease; DVT: deep vein thrombosis; ICD: international classification of disease; PE: pulmonary embolism.

Supplementary Table 3. Covariates included in the propensity score models

	Notes and UK Biobank data field	Baseline set	Imaging set
**Socio-demographics**			
Age (years)	21003	Instance 0	Instance 2
Sex	31		
Ethnicity	21000		
Townsend score	189		
Education	6138	Instance 0	Instance 2
**Physical measurements**			
Systolic blood pressure (mmHg)	Average of automated readings if available (4080), otherwise refer to manual reading (93)	Instance 0	Instance 2
Diastolic blood pressure (mmHg)	Average of automated readings if available (4079), otherwise refer to manual reading (94)	Instance 0	Instance 2
Heart rate (bpm)	Average of automated readings (102) if available, otherwise refer to manual reading (95) – reject heart rates below 40bpm	Instance 0	Instance 2
Body mass index (kg/m^2)	Calculate from height (50) and weight (21002 - or 3160 if not available).	Instance 0	Instance 2
**Laboratory tests**			
HbA1c (mmol/mol)	30750	Instance 0	Instance 0
Random glucose (mmol/L)	30740	Instance 0	Instance 0
Total cholesterol (mmol/L)	30690	Instance 0	Instance 0
HDL (mmol/L)	30760	Instance 0	Instance 0
LDL direct (mmol/L)	30780	Instance 0	Instance 0
Triglyceride level (mmol/L)	30870	Instance 0	Instance 0
**Vascular risk factors**			
Physical activity (METS/week)	As per IPAQ		
Smoking status	20116	Instance 0	Instance 2
Diabetes	As per Table 4	ICD codes until instance 0	ICD codes until instance 2
Hypertension	As per Table 4	ICD codes until instance 0	ICD codes until instance 2
High cholesterol	As per Table 4	ICD codes until instance 0	ICD codes until instance 2

Supplementary Table 3 footnote. Instance 0 indicates baseline visit, instance 2 indicates imaging visit. HbA1c: glycated haemoglobin, HDL: high density lipoprotein, IPAQ: international physical activity questionnaire; LDL: low density lipoprotein; METS: metabolic equivalent.

Supplementary Table 4. ICD and UK Biobank field codes used to define clinical diagnosis of prevalent diabetes, hypertension, and high cholesterol

**Diabetes**		
Self-report	20002	Diabetes
	20002	Type 1 diabetes
	20002	Type 2 diabetes
Medications	6177, 6153: 3	Insulin
ICD9	250	Diabetes mellitus
ICD10	E10	Type 1 diabetes mellitus
	E11	Type 2 diabetes mellitus
	E13	Other specified diabetes mellitus
	E14	Unspecified diabetes mellitus
	G590	Diabetic mononeuropathy
	G632	Diabetic polyneuropathy
	H280	Diabetic cataract
	H360	Diabetic retinopathy
	M142	Diabetic arthropathy
	N083	Glomerular disorders in diabetes mellitus
	O240	Diabetes mellitus in pregnancy: Pre-existing type 1 diabetes mellitus
	O241	Diabetes mellitus in pregnancy: Pre-existing type 2 diabetes mellitus
	O243	Diabetes mellitus in pregnancy: Pre-existing diabetes mellitus, unspecified
	O244	Diabetes mellitus arising in pregnancy
	O249	Diabetes mellitus in pregnancy, unspecified
	Y423	Insulin and oral hypoglycaemic [antidiabetic] drugs
First occurrences	130706	Date E10 first reported (insulin-dependent diabetes mellitus)
	130708	Date E11 first reported (non-insulin-dependent diabetes mellitus)
	130712	Date E13 first reported (other specified diabetes mellitus)
	130714	Date E14 first reported (unspecified diabetes mellitus)
Diagnosed by doctor	2443	Diabetes diagnosed by doctor
	2976	Age diabetes diagnosed by doctor
**High cholesterol**		
Self-report	20002	High cholesterol
Medications	6177, 6153: 1	Cholesterol lowering medication
ICD9	272	Disorders of lipoid metabolism
ICD10	E780	Pure hypercholesterolaemia
	E782	Mixed hyperlipidaemia
	E783	Hyperchylomicronaemia
	E784	Other hyperlipidaemia
	E785	Hyperlipidaemia, unspecified
First occurrences	130814	Date E78 first reported (disorders of lipoprotein metabolism and other lipidaemias)
**Hypertension**		
Self-report	20002	Essential hypertension
	20002	Hypertension
Medications	6177, 6153: 2	Blood pressure medication
First occurrences	131286	Date I10 first reported (essential (primary) hypertension)
Diagnosed by doctor	2966	Age high blood pressure diagnosed
	6150: 4	High blood pressure
ICD10	I10	Essential (primary) hypertension
	I11.0	Hypertensive heart disease with (congestive) heart failure
	I11.9	Hypertensive heart disease without (congestive) heart failure
	I12.0	Hypertensive renal disease with renal failure
	I12.9	Hypertensive renal disease without renal failure
	I13.0	Hypertensive heart and renal disease with (congestive) heart

		failure
	I13.1	Hypertensive heart and renal disease with renal failure
	I13.2	Hypertensive heart and renal disease with both (congestive) heart failure and renal failure
	I13.9	Hypertensive heart and renal disease, unspecified
	I15.0	Renovascular hypertension
	I15.1	Hypertension secondary to other renal disorders
	I15.2	Hypertension secondary to endocrine disorders
	I15.8	Other secondary hypertension
ICD9	4010	Essential hypertension, specified as malignant
	4011	Essential hypertension, specified as benign
	4019	Essential hypertension, not specified as malignant or benign
	4039	Hypertensive renal disease, not specified as malignant or benign

Supplementary Table 4. ICD: international classification of disease

Supplementary Table 5. Number of incident events in the composite haematological cancer category and in subtypes of myeloma, lymphoma, and leukaemia.

	All haem	Myeloma	Lymphoma	Leukaemia
Incident CVDs (N, %)	2032	198	1495	525
IHD	286	27	193	65
NICM	57	2	41	14
HF	227	21	157	47
AF/flutter	259	25	167	66
Stroke	102	11	59	30
Pericarditis	47	3	32	12
VTE (DVT/PE)	102	11	27	63
Mortality outcomes (N, %)	496	109	351	140
Any CVD	47	8	26	12
IHD	26	4	15	7
HF/NICM	5	1	4	0
Stroke	10	1	6	2
Hypertensive diseases	3	1	1	1

Supplementary Table 5 footnote. AF: atrial fibrillation; CVD: cardiovascular disease; IHD: ischaemic heart disease; NICM: non-ischaemic cardiomyopathies; VTE: venous thromboembolism

Supplementary Table 6. Associations with events for those with any haematological cancer and in subtypes of myeloma, lymphoma, and leukaemia- compared to controls

	All haem	Myeloma	Lymphoma	Leukaemia
Incident disease				
IHD	1.96 (1.58-2.43)	1.61 (0.87-2.97)	1.95 (1.52-2.51)	1.97 (1.31-3.00)
	6.0e-10	0.132	1.2e-7	0.001
NICM	2.53 (1.53-4.16)	2.01 (0.18-22.31)	2.29 (1.31-4.01)	3.56 (1.15-10.91)
	0.0003	0.570	0.004	0.03
Heart failure	3.48 (2.61-4.62)	4.44 (1.65-11.97)	3.29 (2.39-4.53)	4.44 (2.29-8.58)
	1.0e-17	0.003	3.1e-13	9.0e-6
AF/flutter	2.00 (1.60-2.50)	1.73 (0.90-3.35)	1.67 (1.30-2.16)	4.10 (2.46-6.82)
	9.4e-10	0.102	0.0001	6.0e-8
Stroke	2.45 (1.68-3.58)	1.39 (0.55-3.53)	1.99 (1.27-3.10)	3.90 (1.80-8.33)
	3.7e-6	0.488	0.002	0.0005
Pericarditis	2.95 (1.64-5.32)	3.02 (0.31-29.24)	2.66 (1.38-5.21)	6.11 (1.36-27.39)
	0.0003	0.341	0.003	0.02
VTE	2.69 (1.80-4.00)	2.83 (0.89-9.07)	2.92 (1.77-4.76)	2.23 (1.13-4.35)
	1.2e-6	0.079	0.00003	0.02
Mortality outcomes				
All-cause	3.78 (3.17-4.52)	7.74 (4.82-12.44)	3.78 (3.06-4.66)	3.67 (2.64-5.05)
	7.5e-49	2.9e-17	8.0e-35	7.8e-15
Any CVD	1.26 (0.79-2.01)	8.10 (1.00-65.85)	1.00 (0.58-1.73)	2.46 (0.91-6.62)
	0.329	0.05	0.99	0.07
IHD	1.58 (0.80-3.09)	4.01 (0.44-36.35)	1.25 (0.58-2.67)	3.53 (0.73-16.95)
	0.186	0.217	0.57	0.12
Heart failure or NICM	0.81 (0.22-3.01)	-	0.80 (0.21-3.00)	-
	0.749	-	0.74	-
Stroke	1.01 (0.40-2.54)	-	1.00 (0.32-3.11)	-
	0.988	-	0.999	-
Hypertensive diseases	-	-	-	-
	-	–	-	-

Supplementary Table 6 footnote. AF: atrial fibrillation; CVD: cardiovascular disease; IHD: ischaemic heart disease; NICM: non-ischaemic cardiomyopathies; VTE: venous thromboembolism

Supplementary Table 7. Incident events observed by cancer site (including all prevalent cancers, without covariate imputation – only those with complete data)

Complete cases	Breast	Lung	Prostate	Colorectal	Uterus	Haem	Total
**Incident disease**							
IHD	307	21	241	142	32	188	931
NIC	42	1	19	22	2	28	114
HF	156	14	121	71	17	138	517
AF/flutter	272	18	245	141	36	157	869
Stroke	101	8	90	44	11	62	316
Pericarditis	45	7	16	16	3	28	115
VTE	148	7	89	47	14	62	367
**Mortality outcomes**							
All-cause	693	82	419	290	46	339	1869
CVD (any)	10	5	31	20	3	17	86
IHD	6	0	5	4	3	2	20
HF/NIC	11	1	12	2	0	9	35
Stroke	2	0	3	2	1	1	9
Hypertensive diseases	33	7	53	27	6	32	158

Supplementary Table 7 footnote. AF: atrial fibrillation; CVD: cardiovascular disease; DVT: deep vein thrombosis; HF: heart failure; IHD: ischaemic heart disease; NICM: non-ischaemic cardiomyopathies; PE: pulmonary embolism.

Supplementary Table 8. Associations of cancer with incident events amongst all prevalent cancers with complete data (no imputation)

	Breast	Lung	Prostate	Colorectal	Uterus	Haem
**Incident disease**						
IHD	1.13 (0.96, 1.34)	1.42 (0.70, 2.89)	1.01 (0.84, 1.22)	1.08 (0.85, 1.38)	0.90 (0.54, 1.51)	1.88 (1.48, 2.41)
	0.141	0.326	0.926	0.51	0.705	3.22×10^{-7}
NICM	1.75 (1.06, 2.92)	–	0.90 (0.49, 1.68)	2.20 (1.07, 4.57)	–	3.53 (1.60, 7.77)
	0.028	–	0.754	0.033	–	0.002
HF	1.31 (1.03, 1.67)	2.05 (0.76, 5.58)	0.94 (0.74, 1.21)	1.21 (0.85, 1.70)	1.62 (0.73, 3.60)	2.18 (1.62, 2.94)
	0.028	0.159	0.653	0.285	0.244	2.75×10^{-7}
AF/flutter	1.11 (0.93, 1.31)	1.32 (0.62, 2.86)	0.91 (0.76, 1.09)	1.34 (1.04, 1.70)	1.26 (0.78, 2.05)	1.79 (1.38, 2.29)
	0.986	0.466	0.343	0.023	0.346	9.00×10^{-6}
Stroke	1.00 (0.76, 1.31)	1.15 (0.44, 3.00)	0.83 (0.63, 1.11)	0.79 (0.52, 1.19)	0.92 (0.40, 2.12)	2.89 (1.77, 4.76)
	0.986	0.783	0.206	0.248	0.844	2.67×10^{-5}
Pericarditis	1.84 (1.12, 3.03)	2.36 (0.61, 9.30)	0.80 (0.41, 1.55)	2.69 (1.04, 6.89)	–	3.13 (1.48, 6.69)
	0.017	0.215	0.508	0.04	–	0.003
VTE (DVT/PE)	1.62 (1.23, 2.1)	1.51 (0.41, 5.47)	1.20 (0.89, 1.62)	1.02 (0.67, 1.55)	1.07 (0.53, 2.18)	2.34 (1.49, 3.71)
	0.0005	0.537	0.243	0.927	0.841	0.0002
**Mortality outcomes**						
All-cause	2.35 (2.01, 2.90)	6.40 (0.79, 10.80)	1.65 (1.41, 1.92)	2.31 (1.88, 2.83)	2.18 (1.31, 3.62)	3.77 (3.01, 4.70)
	5.80×10^{-36}	3.65×10^{-12}	1.66×10^{-10}	8.28×10^{-16}	0.003	1.43×10^{-31}
Any CVD	0.90 (0.56, 1.43)	1.75 (0.50, 6.11)	0.91 (0.63, 1.34)	1.28 (0.73, 2.25)	2.01 (0.50, 8.08)	1.60 (0.92, 2.80)
	0.638	0.378	0.636	0.392	0.321	0.092
IHD	0.63 (0.28, 1.38)	2.51 (0.48, 13.2)	0.72 (0.45, 1.15)	1.67 (0.81, 3.39)	–	1.00 (0.51, 1.97)
	0.245	0.278	0.166	0.168	–	0.997
HF/NIC	2.01 (0.50, 8.00)	–	1.67 (0.40, 6.96)	–	–	–
	0.323	–	0.484	–	–	–
Stroke	0.84 (0.38, 1.90)	–	1.92 (0.662, 4.35)	–	–	9.03 (1.14, 71.52)
	0.686	–	0.258	–	–	0.037
Hypertensive diseases	–	–	–	–	–	–
	–	–	–	–	–	–

Supplementary Table 8 footnote. Results are sub-distribution hazard ratio (95% confidence interval) and p-value associated with cancer exposure (vs no cancer). Comparators are matched on age, sex, ethnicity, deprivation, education, blood pressure, heart rate, body mass index, glycated haemoglobin, random glucose, total cholesterol, high density lipoprotein, low density lipoprotein, triglyceride level, physical activity, smoking, diabetes, hypertension, and high cholesterol. AF: atrial fibrillation; CVD: cardiovascular disease; IHD: ischaemic heart disease; NICM: non-ischaemic cardiomyopathies; VTE: venous thromboembolism. AF: atrial fibrillation; CVD: cardiovascular disease; DVT: deep vein thrombosis; HF: heart failure; IHD: ischaemic heart disease; NIC: non-ischaemic cardiomyopathies; PE: pulmonary embolism.

Supplementary Table 9. Associations of cancer with incident cardiovascular events compared to matched controls (cause specific hazard ratios)

	Breast	Lung	Prostate	Colorectal	Uterus	Haematological
**Incident disease**						
IHD	1.12 (0.99, 1.26)	1.43 (0.95, 2.18)	0.97 (0.84, 1.13)	**1.23 (1.02, 1.49)**	1.06 (0.77, 1.48)	**2.14 (1.75, 2.61)**
	0.085	0.089	0.712	**0.032**	0.697	1.40×10^{-13}
NICM	**1.92 (1.36, 2.72)**	–	1.22 (0.76, 1.97)	1.36 (0.79, 2.34)	3.63 (0.76, 17.64)	**2.89 (1.77, 4.71)**
	0.0002	–	0.399	0.257	0.107	0.00002
Heart failure	**1.42 (1.21, 1.68)**	**2.59 (1.45, 4.66)**	1.11 (0.90, 1.34)	0.84 (0.66, 1.08)	1.42 (0.90, 2.27)	**4.01 (3.03, 5.26)**
	0.00002	0.001	0.343	0.187	0.138	3.10×10^{-23}
AF/flutter	**1.17 (1.04, 1.32)**	**1.88 (1.14, 3.13)**	1.06 (0.91, 1.22)	**1.36 (1.13, 1.65)**	1.03 (0.73, 1.46)	**2.20 (1.79, 2.72)**
	**0.011**	**0.014**	0.451	0.002	0.846	1.4×10^{-13}
Stroke	1.20 (0.97, 1.46)	1.72 (0.83, 3.60)	1.25 (0.98, 1.57)	1.21 (0.89, 1.67)	1.20 (0.71, 2.01)	**2.53 (1.80, 3.60)**
	0.087	0.150	0.078	0.219	0.498	1.6×10^{-7}
Pericarditis	**2.14 (1.45, 3.19)**	**16.78 (2.16, 131.63)**	1.23 (0.71, 2.14)	1.48 (0.74, 2.94)	3.63 (0.75, 17.46)	**3.35 (1.90, 5.99)**
	0.0002	0.007	0.454	0.270	0.109	0.00003
VTE	**1.52 (1.27, 1.82)**	1.54 (0.73, 3.25)	**1.79 (1.36, 2.32)**	1.30 (0.94, 1.80)	1.75 (0.94, 3.29)	**3.03 (2.08, 4.39)**
	4.60×10^{-6}	0.263	0.00003	0.114	0.076	7.9×10^{-9}
**Mortality outcomes**						
All-cause	**2.48 (2.25, 2.72)**	**5.00 (3.63, 6.89)**	**1.65 (1.46, 1.86)**	**2.08 (1.79, 2.41)**	**2.41 (1.73, 3.32)**	**4.14 (3.49, 4.90)**
	3.65×10^{-80}	7.25×10^{-21}	2.40×10^{-16}	1.30×10^{-21}	3.06×10^{-7}	3.10×10^{-59}
Any CVD	1.04 (0.76, 1.43)	**3.49 (1.43, 8.50)**	0.93 (0.69, 1.26)	1.31 (0.88, 1.95)	1.26 (0.59, 2.69)	**1.70 (1.08, 2.66)**
	0.809	**0.006**	0.646	0.181	0.553	**0.022**
IHD	0.68 (0.40, 1.13)	**2.94 (1.16, 7.39)**	0.93 (0.64, 1.35)	1.16 (0.71, 1.88)	–	**1.99 (1.04, 3.78)**
	0.131	**0.02**	0.693	0.551	–	0.036
Heart failure or NICM	**9.12 (2.10, 39.65)**	–	0.83 (0.31, 2.23)	5.42 (0.64, 45.50)	–	1.17 (0.34, 4.10)
	0.003	–	0.708	0.121	–	0.797
Stroke	0.93 (0.52, 1.68)	–	1.00 (0.51, 1.99)	1.35 (0.56, 3.25)	5.00 (0.58, 42.95)	1.28 (0.52, 3.22)
	0.816	–	0.995	0.498	0.142	0.587
Hypertensive diseases	**8.58 (1.07, 68.72)**	–	1.34 (0.36, 5.00)	–	**–**	–
	0.043	**–**	0.668	–	**–**	–

Supplementary Table 9 footnote. Results are cause specific hazard ratio (95% confidence interval) and p-value associated with cancer history (vs no cancer). Comparators are matched on age, sex, ethnicity, deprivation, education, blood pressure, heart rate, body mass index, glycated haemoglobin, random glucose, total cholesterol, high density lipoprotein, low density lipoprotein, triglyceride level, physical activity, smoking, diabetes, hypertension, and high cholesterol. AF: atrial fibrillation; CVD: cardiovascular disease; IHD: ischaemic heart disease; NICM: non-ischaemic cardiomyopathies; VTE: venous thromboembolism.

Supplementary Table 10. Incident events observed by cancer site (including cancers within preceding 5 years)

within 5 years	Breast	Lung	Prostate	Colorectal	Uterus	Haem	Total
**Incident disease**							
IHD	197	17	273	136	29	99	751
NIC	33	0	22	15	4	21	95
HF	120	15	133	55	15	85	423
AF/flutter	178	22	261	120	25	120	726
Stroke	69	14	105	36	11	37	272
Pericarditis	27	6	20	10	3	17	83
VTE (DVT/PE)	129	14	108	43	10	47	351
**Mortality outcomes**							
All-cause	594	124	502	321	56	309	1906
CVD (any)	30	9	58	25	7	20	149
IHD	15	7	38	19	2	14	95
HF/NIC	4	0	6	2	2	0	14
Stroke	7	1	10	3	2	6	29
Hypertensive diseases	2	0	4	2	2	0	10

Supplementary Table 10 footnote. AF: atrial fibrillation; CVD: cardiovascular disease; DVT: deep vein thrombosis; HF: heart failure; IHD: ischaemic heart disease; NIC: non-ischaemic cardiomyopathies; PE: pulmonary embolism.

Supplementary Table 11. Associations of cancer with incident events amongst all prevalent cancers for cases diagnoses in the preceding 5 years

	Breast	Lung	Prostate	Colorectal	Uterus	Haem
**Incident disease**						
IHD	1.15 (0.93, 1.40)	0.93 (0.45, 1.92)	0.94 (0.79, 1.13)	1.12 (0.86, 1.45)	0.76 (0.47, 1.21)	1.16 (0.85, 1.57)
	0.195	0.843	0.518	0.399	0.245	0.347
NICM	1.84 (1.03, 3.29)	–	0.70 (0.41, 1.22)	1.07 (0.52, 2.25)	4.01 (0.44, 36.23)	2.64 (1.16, 5.99)
	0.038	–	0.213	0.847	0.215	0.02
HF	1.52 (1.14, 2.01)	1.57 (0.66, 3.71)	0.94 (0.79, 1.19)	0.79 (0.55, 1.11)	0.75 (0.39, 1.43)	2.12 (1.48, 3.06)
	0.004	0.302	0.592	0.17	0.378	5.35×10^{-5}
AF/flutter	0.98 (0.79, 1.21)	1.65 (0.84, 3.25)	0.94 (0.79, 1.12)	1.02 (0.79, 1.32)	0.76 (0.44, 1.27)	1.68 (1.25, 2.27)
	0.859	0.153	0.485	0.864	0.29	0.001
Stroke	0.90 (0.65, 1.26)	1.57 (0.66, 3.71)	1.06 (0.80, 1.40)	1.06 (0.66, 1.72)	0.79 (0.35, 1.75)	1.92 (1.11, 3.35)
	0.567	0.307	0.67	0.79	0.56	0.021
Pericarditis	3.03 (1.42, 6.42)	6.05 (0.73, 50.91)	1.54 (1.13, 2.10)	0.59 (0.27, 1.30)	–	2.44 (1.00, 5.87)
	0.004	0.096	0.006	0.186	–	0.049
VTE (DVT/PE)	1.90 (1.40, 2.53)	2.89 (1.08, 7.69)	1.54 (1.13, 2.10)	1.19 (0.75, 1.86)	0.71 (0.34, 1.51)	2.14 (1.28, 3.53)
	2.14×10^{-5}	0.034	0.006	0.47	0.375	0.004
**Mortality outcomes**						
All-cause	3.40 (2.89, 4.01)	6.12 (4.1, 9.14)	1.69 (1.47, 1.95)	3.20 (2.58, 3.96)	1.55 (1.03, 2.34)	4.48 (3.50, 5.72)
	1.56×10^{-48}	6.69×10^{-19}	2.14×10^{-13}	1.13×10^{-26}	0.037	3.55×10^{-33}
Any CVD	0.97 (0.58, 1.60)	1.12 (0.42, 2.97)	1.12 (0.76, 1.63)	1.26 (0.70, 2.27)	1.19 (0.4, 3.53)	1.43 (0.73, 2.77)
	0.898	0.824	0.56	0.449	0.761	0.298
IHD	1.00 (0.49, 2.03)	1.39 (0.43, 4.48)	1.16 (0.73, 1.86)	1.27 (0.64, 2.51)	–	1.39 (0.62, 3.16)
	0.997	0.578	0.541	0.491	–	0.424
HF/NIC	–	–	2.01 (0.50, 8.00)	–	–	–
	–	–	0.326	–	–	–
Stroke	0.70 (0.27, 1.84)	–	0.83 (0.36, 1.92)	–	–	3.00 (0.61, 14.88)
	0.472	–	0.83	–	–	0.179
Hypertensive diseases	–	–	–	–	–	–
	–	–	–	–	–	–

Supplementary Table 11 footnote. Results are sub-distribution hazard ratio (95% confidence interval) and p-value associated with cancer exposure (vs propensity-matched non-cancer controls). AF: atrial fibrillation; CVD: cardiovascular disease; DVT: deep vein thrombosis; HF: heart failure; IHD: ischaemic heart disease; NIC: non-ischaemic cardiomyopathies; PE: pulmonary embolism.

Supplementary Table 12. Characteristics of the imaging subset

	Cases	Controls	Breast	Lung	Prostate	Colorectal	Uterus	Haem
N	1354	1354	586	13	473	47	76	159
Age	68 [62-72]	68 [62-72]	66 [59-70]	68 [64-69]	70 [66-73]	67 [62-71]	66 [59-70]	67 [62-72]
Men	603 (44.5)	609 (45.0)	0 (0)	4 (30.8)	473 (100)	29 (61.7)	0 (0)	61.0 (97)
Women	751 (55.5)	745 (55.0)	586 (100)	9 (69.2)	0 (0)	18 (38.3)	76 (100)	62 (39.0)
White ethnicity	1329 (98.2)	1333 (98.6)	576 (98.3)	13 (100)	466 (98.5)	45 (95.7)	74 (97.4)	155 (98.1)
BAME	24 (1.8)	19 (1.4)	10 (1.7)	0 (0)	7 (1.5)	2 (4.3)	2 (2.6)	3 (1.9)
Townsend score	-2.8 [-4.0, -0.9]	-2.8 [-4.0, -0.7]	-2.8 [-3.9, -0.9]	-3.3 [-4.3, -2.7]	-3.0 [-4.1, -1.5]	-2.5 [-4.0, -0.8]	-2.6 [-3.6, -0.9]	-2.5 [-3.8, 0.0]
Degree or professional qualification	882 (65.3)	879 (65.0)	377 (64.4)	4 (30.8)	316 (67.0)	36 (76.6)	49 (64.5)	100 (63.3)
SBP (mmHg)	138.9 ±17.9	139.2 ±18.8	135.3 ± 18.3	133.9 ± 13.1	142.7 ±16.1	142.2 ±18.8	137.8 ± 19.8	140.1 ±18.0
DBP (mmHg)	77.9 ±9.5	77.8 ± 9.8	77.1 ±9.4	79.9 ±7.5	78.4 ±9.3	80.2 ±9.1	78.6 ±9.5	78.2 ±10.4
HR (bpm)	70 [62.5-79.5]	71 [63-79.5]	72.5 [65-81.5]	73.5 [62-86]	67 [60-76]	69.8 [61-81]	70.5 [64.5-78]	69.5 [62-79]
BMI (kg/m^2)	26.0 [23.4 – 29.0]	25.9 [23.5-28.6]	25.2 [22.7-28.6]	26.7 [23.1-29.3]	26.5 [24.5-28.9]	27.1 [24.4- 31.8]	27.7 [24.2- 31.3]	25.5 [22.9- 28.6]
Physical activity (METS/week)	2026 [1006-3566]	1939 [938-3492]	2179 [1009-3546]	1701 [1026-3572]	1983 [1045-3594]	1194 [718-2549]	1662 [974-3512]	2039 [1055 – 4086]
Ever Smoking	508 (38.3)	532 (40.0)	210 (36.6)	7 (53.9)	195 (42.1)	22 (46.8)	19 (25.3)	55 (35.3)
HbA1c (mmol/mol)	35.1 [32.8- 37.4]	35.2 [32.5- 37.5]	35 [32.7- 37.1]	34.2 [33.1-37.7]	35.4 [33.1-37.5]	35 [32.4- 38.3]	36.0 [33.5- 38.7]	34.8 [32.5-37.2]
Random glucose (mmol/L)	4.9 [4.6- 5.3]	4.9 [4.6- 5.3]	4.9 [4.6- 5.3]	5.0 [4.1- 5.6]	4.9 [4.6- 5.3]	5.0 [4.7- 5.5]	4.9 [4.5- 5.2]	4.9 [4.5- 5.2]

	Cases	Controls	Breast	Lung	Prostate	Colorectal	Uterus	Haem
Total cholesterol (mmol/L)	5.7 ±1.2	5.7 ±1.1	5.9 ±1.2	6.1 ±1.1	5.4 ±1.1	5.5 ±1.4	5.8 ±1.1	5.4 ±1.1
HDL (mmol/L)	1.4 [1.2- 1.7]	1.4 [1.2- 1.7]	1.6 [1.3- 1.8]	1.5 [1.2- 1.6]	1.2 [1.1- 1.5]	1.3 [1.0- 1.5]	1.4 [1.3- 1.8]	1.3 [1.1- 1.6]
LDL direct (mmol/L)	3.5 [3.0- 4.1]	3.5 [2.9- 4.1]	3.6 [3.0- 4.2]	3.7 [3.2- 4.4]	3.4 [2.9- 4.0]	3.4 [2.8- 4.0]	3.4 [3.0- 4.1]	3.4 [2.9-4.0]
Triglyceride level (mmol/L)	1.5 [1.1- 2.1]	1.5 [1.0- 2.1]	1.4 [1.0- 1.9]	1.4 [1.4- 2.4]	1.7 [1.2- 2.4]	1.6 [1.3- 2.2]	1.5 [0.9- 1.9]	1.5 [1.0- 2.2]
Diabetes	109 (8.1)	121 (8.9)	34 (5.8)	1 (7.7)	47 (9.9)	8 (17.0)	11 (14.5)	8 (5.0)
Hypertension	505 (37.3)	485 (35.8)	160 (27.3)	4 (30.8)	230 (48.6)	24 (51.1)	26 (34.2)	61 (38.4)
High cholesterol	618 (45.6)	631 (46.6)	206 (35.2)	7 (53.9)	280 (59.2)	30 (63.8)	30 (39.5)	65 (40.9)
LVM (g)	-	-	70.9 ± 12.8	80.4 ± 17.8	99.7 ± 17.4	92.4 ± 21.3	75.3 ± 18.0	91.1 ± 25.7
LVEDV (ml)	-	-	128.6 ± 22.1	131.7 ± 34.1	163.2 ± 32.1	150.0 ± 33.5	133.4 ±27.5	155.4 ± 42.2
LVEF (%)	-	-	60.5 ± 6.2	62.2 ± 3.3	57.8 ± 6.6	59.3 ± 4.5	61.8 ± 5.1	57.3 ± 6.7
LVGFI (%)	-	-	0.50 ± 0.07	0.49 ± 0.05	0.45 ± 0.07	0.46 ± 0.06	0.50 ± 0.06	0.45 ± 0.07
LV GLS (%)	-	-	-19.1 ± 3.0	-19.5 ± 2.2	-17.7 ± 2.6	-17.7 ± 2.5	-19.4 ± 2.4	-17.4 ± 2.7
LAV max (ml)	-	-	66.9 ± 19.4	62.8 ± 26.0	77.3 ± 29.7	72.3 ± 22.5	71.4 ± 22.7	77.4 ± 29.6
LAEF (%)	-	-	61.6 ± 9.1	62.5 ±14.3	59.1 ± 10.6	61.2 ± 8.5	61.4 ± 7.5	58.3 ± 10.8

Supplementary Table 12 footnote. Continuous variables are shown as mean ±standard deviation, or median [IQR] if skewed. Count variables are shown as N (%). BAME: Black, Asian, and Minority ethnic; HbA1c: glycated haemoglobin, HDL: high density lipoprotein, LDL: low density lipoproteinDBP: diastolic blood pressure; METS: metabolic equivalent of task; SBP: systolic blood pressure. LA: left atrium; LV: left ventricle; LA ejection fraction (LAEF); LA maximum volume (LAV); LV end-diastolic volume (LVEDV); LV mass (LVM); LV ejection fraction (LVEF); LV global function index (LVGFI); LV global longitudinal strain (GLS).

Supplementary Table 13. Association of cancer with CMR metrics in participants without cardiovascular disease at time of imaging

	**Breast**	**Lung**	**Prostate**	**Colorectal**†	**Uterus**†	**Haem**†
**LVM (g)**	0.05 (-0.08, 0.18)	-0.55 (-1.52, 0.41)	-0.12 (-0.29, 0.06)	-0.69 (-1.36, -0.02)	0.16 (-0.22, 0.54)	0.08 (-0.20, 0.37)
	0.44	0.22	0.19	0.04	0.40	0.56
**LVEDV (ml)**	0.07 (-0.05, 0.19)	-0.69 (-1.96, 0.58)	-0.07 (-0.23, 0.10)	-0.88 (-1.60, -0.15)	0.14 (-0.25, 0.53)	0.19 (-0.10, 0.47)
	0.26	0.24	0.42	0.02	0.47	0.20
**LVEF (%)**	-0.17 (-0.30, -0.04)	0.24 (-1.07, 1.55)	0.12 (-0.04, 0.29)	0.33 (-0.62, 1.29)	0.15 (-0.23, 0.53)	-0.26 (-0.52, -0.00)
	0.01	0.68	0.14	0.47	0.42	0.05
**LVGFI (%)**	-0.14 (-0.27, -0.01)	-0.08 (-1.72, 1.56)	0.13 (-0.04, 0.3)	0.22 (-0.74, 1.18)	0.16 (-0.24, 0.56)	-0.17 (-0.42, 0.08)
	0.04	0.91	0.13	0.64	0.42	0.19
**LV GLS (%)**	-0.02 (-0.15, 0.10)	-0.43 (-1.72, 0.86)	0.03 (-0.14, 0.21)	-0.11 (-1.11, 0.89)	-0.00 (-0.37, 0.37)	0.19 (-0.09, 0.47)
	0.74	0.45	0.71	0.82	0.99	0.18
**LAV max (ml)**	0.09 (-0.04, 0.21)	-1.10 (-2.28, 0.08)	-0.03 (-0.21, 0.15)	-0.92 (-1.80, 0.05)	0.21 (-0.13, 0.55)	0.21 (-0.07, 0.48)
	0.18	0.06	0.74	0.04	0.23	0.13
**LAEF (%)**	-0.15 (-0.27, -0.02)	0.59 (0.03, 1.15)	-0.01 (-0.18, 0.16)	0.54 (-0.12, 1.21)	-0.07 (-0.38, 0.24)	-0.324(-0.60, -0.05)
	0.03	0.04	0.90	0.10	0.66	0.02

Supplementary Table 13 footnote. The results are standardised beta-coefficients and 95% confidence intervals, thus representing standard deviation change in CMR metrics with change in cancer exposure status from non-cancer to cancer; for standard deviation of each metric please refer to Supplementary Table 5. The bold and yellow shaded cells represent statistically significant associations. LA: left atrium; LV: left ventricle; LV end-diastolic volume (LVEDV), LV mass (LVM), LVM: LVEDV, LV stroke volume (LVSV), LV ejection fraction (LVEF), LV global function index (LVGFI), LV global longitudinal strain (GLS), LA maximum volume (LAV), LA ejection fraction (LAEF).
